# DMAP1 Deficiency Suppresses Lung Cancer Progression by Destabilizing Replication Fork and Activating IFN Signaling‐Mediated Anti‐tumor Immunity

**DOI:** 10.1002/advs.202517634

**Published:** 2026-03-29

**Authors:** Kan Huang, Xi Dai, Shuaihu Li, Yingxue Chen, Yaxin Yu, Lin Wang, Kun Liu, Shuhan Lyu, Chongyang Li, Yihua Sun, Fei Li

**Affiliations:** ^1^ Department of pathology, School of Basic Medical Sciences Department of Thoracic Surgery, Fudan University Shanghai Cancer Center Fudan University Shanghai China; ^2^ Department of Thoracic Surgery and State Key Laboratory of Genetic Engineering Fudan University Shanghai Cancer Center Shanghai China; ^3^ Institute of Thoracic Oncology Fudan University Shanghai China; ^4^ Department of Oncology Shanghai Medical College Fudan University Shanghai China; ^5^ Department of Pathology and Frontier Innovation Center School of Basic Medicine Sciences, Shanghai Pudong Hospital Fudan University Shanghai China; ^6^ Department of Medicine Keck School of Medicine University of Southern California Los Angeles USA; ^7^ Department of Medical Oncology Shanghai Pulmonary Hospital School of Medicine Tongji University Shanghai China; ^8^ Department of Thoracic Surgery Shanghai Chest Hospital Shanghai Jiao Tong University School of Medicine Shanghai China; ^9^ Shanghai Institute of Thoracic Oncology Shanghai China

**Keywords:** DMAP1, IFN signaling, lung cancer, replication stress, tumor immunity

## Abstract

Despite substantial progress in targeted and immune therapies, lung cancer remains the leading cause of cancer‐related mortality, highlighting the urgent need for novel therapeutic strategies. Through a CRISPR‐based knock‐out screen, we identified the DNA methyltransferase 1‐associated protein 1 (DMAP1) as a critical regulator of lung cancer progression. Functional studies demonstrated that DMAP1 deficiency exerts its anti‐tumor effects through attenuating tumor cell proliferation and activating T cell‐mediated adaptive anti‐tumor effects. Mechanistically, DMAP1 deficiency causes replication fork retardance, disturbs genome stability, and induces endogenous DNA damage, thereby activating IFN signaling‐mediated anti‐tumor immune response. Clinical data analyses revealed that high DMAP1 expression is associated with a “cold” tumor microenvironment and poorer overall survival in lung cancer. These findings significantly advance our knowledge of DMAP1's function in lung cancer development and offer a scientific basis for designing novel treatment approaches.

## Graphical abstract

Lung cancer remains the leading cause of cancer‐related death. We investigated the role of the epigenetic regulator DMAP1 in NSCLC and found that its loss induces replication stress and DNA damage. This in turn activates type I IFN signaling via the cGAS–STING pathway and transcriptional ISG de‐repression, enhancing anti‐tumor immune responses.

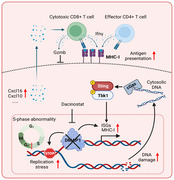

## 1 Introduction

Lung cancer remains the leading cause of cancer‐related deaths globally [1]. Among its subtypes, non‐small cell lung cancer (NSCLC) accounts for approximately 85% of cases and has achieved significant therapeutic advances, including the development of targeted therapies and immune checkpoint blockade (ICB) [2]. However, despite the clinical success of small‐molecule inhibitors and PD‐1/PD‐L1 blockade in certain patient subsets, their overall efficacy remains limited. Many patients either fail to respond initially or eventually develop resistance, contributing to persistently poor long‐term outcomes [3, 4]. Consequently, the five‐year survival rate for lung cancer remains unacceptably low, underscoring the critical need for novel therapeutic targets and strategies to improve immune responsiveness.

Epigenetic regulators critically modulate cellular states and drive tumor evolution. Their frequent dysregulation in cancers, including lung cancer, underscores their potential as promising therapeutic targets [5, 6]. Many small‐molecule inhibitors have been developed to target various epigenetic processes, such as DNMT inhibitors for DNA methylation, EZH2 inhibitors for histone remodeling, and LSD inhibitors for histone modifications [7, 8, 9]. However, these drugs have shown limited effectiveness in solid tumors, with most success observed in hematologic malignancies. Notably, recent studies in solid tumors suggest that targeting epigenetic factors, including *FBXO44* and *SMARCAL1*, can induce genome instability and DNA damage response (DDR), generating cytosolic double‐strand DNA (dsDNA) and activating tumor‐intrinsic immune responses [10, 11]. This signaling cascade is initiated by pattern‐recognition receptors (PRRs), such as cGAS, which detects cytosolic DNA, leading to STING activation, and IRF3/7‐dependent transcription of type I interferons (IFNs) and IFN stimulatory genes (ISGs). Through the ensuing IFN signaling, these molecules promote antigen presentation, enhance cytotoxic T cell activation, and facilitate immune‐mediated tumor rejection within the tumor microenvironment [12, 13].

DNA methyltransferase 1‐associated protein 1 (DMAP1) was first identified as a transcriptional co‐repressor that localizes to replication foci through its interaction with DNMT1 during the S phase [14]. However, its role in cancer progression remains controversial and context‐dependent. In gastric cancer and neuroblastoma, DMAP1 suppresses tumor growth via ATM/P53 activation, although this effect is lost in p53‐mutant cells [15, 16]. In pancreatic ductal adenocarcinoma (PDAC), DMAP1 promotes cancer cell apoptosis through interaction with BUB3 and silencing of anti‐apoptotic genes [17]. Additionally, disrupting the DNMT1‐DMAP1 interaction does not affect tumor progression but enhances sensitivity to chemotherapy and radiotherapy in mouse glioma models [18]. Its role in lung cancer progression and immune modulation, however, has yet to be investigated.

Given the therapeutic potential of targeting epigenetic regulators in lung cancer, in this study, we systematically investigated their functional roles in lung cancer progression. Utilizing epigenetic CRISPR loss‐of‐function screens in a murine KP (Kras^G12D^, Trp53^−/−^) lung adenocarcinoma (LUAD) model, we identified a novel vulnerability in lung cancer, DMAP1, targeting which both retarded tumor growth and activated host anti‐tumor immunity. These insights revealed a novel function of DMAP1 and elucidated its mechanistic role in shaping the tumor immune microenvironment, offering valuable guidance for the development of new therapeutic strategies in lung cancer.

## 2 Results

### 2.1 Epigenetic CRISPR Screening Identifies Dmap1 as a Critical Regulator of Lung Cancer Progression

**FIGURE 1:**
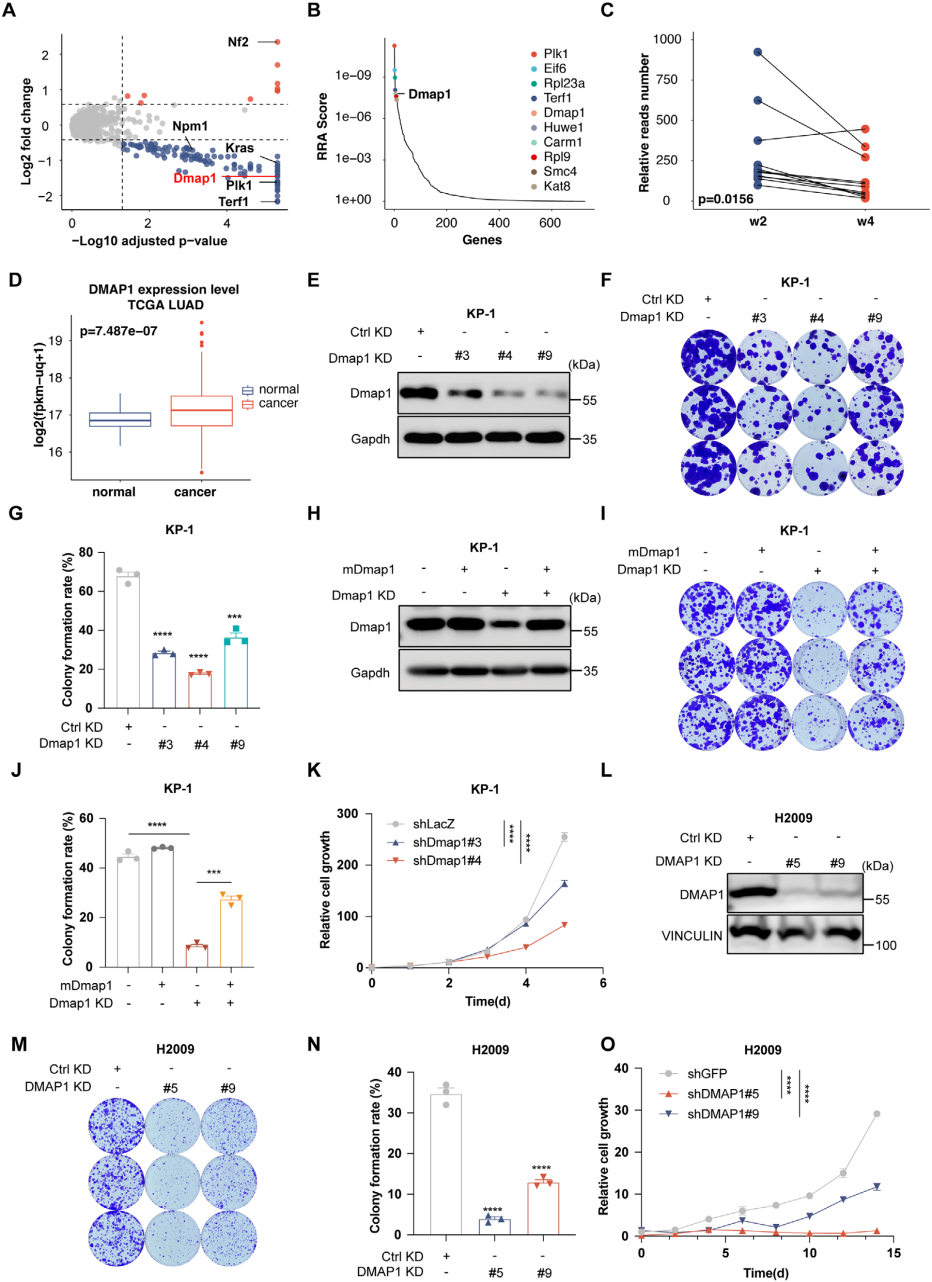
Epigenetic CRISPR screening identifies Dmap1 as a critical gene for lung cancer progression. (A) Volcano plot of comparison between the week 2 and the week 4 clone 7 KP‐1 cells from the screen. (B) RRA plot for the top 10 candidates of the screen in clone 7 KP‐1 cells. (C) Performance of sgRNAs targeting Dmap1 in clone 7 KP‐1 cells of the screen, *n* = 11. (D) The comparison of DMAP1 expression in human normal lung and LUAD samples. Data is obtained from the TCGA LUAD cohort in the UCSC Xena database, *n* = 587. (E) Western blot of Dmap1 knockdown (shDmap1) in KP‐1 cells. (F) Colony formation assays of KP‐1‐shLacZ (Ctrl KD) or KP‐1‐shDmap1 (Dmap1 KD) cells. (G) Statistical analysis for (F), *n* = 3. (H) Western blot for Dmap1 rescue in KP‐1 cells. (I) Colony formation assays of Dmap1 rescue in KP‐1 cells. (J) Statistical analysis for (I) *n* = 3. (K) Cell viability of KP‐1 shLacZ or shDmap1 cells was measured by CCK‐8 assay using a doxycycline (Dox)‐inducible knockdown system, *n* = 5. (L) Western blot of DMAP1 knockdown in H2009 cells. (M) Colony formation assays of H2009‐shGFP (Ctrl KD) or shDMAP1 (DMAP1 KD) cells. (N) Statistical analysis for (M), *n* = 3. (O) Cell viability of H2009 shGFP or H2009 shDMAP1 cells was measured by CCK‐8 assay, *n* = 3. Data are presented as mean ± SEM. Data were analyzed using paired *t*‐test [(C)], Welch's *t*‐test [(D)], and *t*‐test [(G), (J), (K), (N), and (O)]. ^***^, *p* < 0.001; ^****^, *p* < 0.0001. Figure 1A–C was reproduced with permission [21].

To uncover novel epigenetic regulators involved in lung cancer progression, we performed pooled CRISPR‐Cas9 loss‐of‐function screens as previously mentioned (Figure S1A) [19, 20]. Two distinct KP‐Cas9 clones, clone 7 and clone 9, were utilized in the screens, and Npm1, which was previously identified as a therapeutic vulnerability in NSCLC, ranked among the top candidates in both clones (Figure 1A; Figure S1B) [20]. Notably, the screen also highlighted several underexplored epigenetic factors, including Dmap1 (Figure 1A–C; Figure S1B–D).

To further investigate the clinical relevance of Dmap1 in lung cancer, we analyzed data from the Cancer Genome Atlas (TCGA) LUAD cohort. Among 528 LUAD patients, DMAP1 expression was significantly elevated in tumor tissues compared to adjacent normal tissues (Figure 1D). Consistently, the Human Protein Atlas (HPA) also showed markedly higher DMAP1 protein levels in lung cancer tissues (Figure S1E, F), suggesting a potential role for DMAP1 upregulation in lung cancer progression.

### 2.2 Dmap1 Knockdown Suppresses Lung Cancer Progression

To further examine the role of DMAP1 in lung cancer progression, we used KP (Kras^G12D/+^, Trp53^−/−^) LUAD cell line KP‐1 to generate Dmap1‐knockdown (KD) cells using short hairpin RNA (shRNA) (Figure 1E). Consistent with our screen results, Dmap1‐deficient cells showed significantly reduced colony formation capacity (Figure 1F, G). To rule out off‐target effects, re‐expression of shRNA‐resistant Dmap1 restored the clonogenic capacity of Dmap1‐KD cells (Figure 1H–J). CCK‐8 assays further confirmed impaired cell viability upon Dmap1 depletion (Figure 1K). To further validate the function of DMAP1 in human lung cancer, we transduced DMAP1‐knockdown constructs into two widely used human LUAD cell lines, H2009 and A549, both of which exhibit modest endogenous DMAP1 expression (Figure 1L; Figure S2A, B). As expected, DMAP1 knockdown significantly impaired proliferation and viability in both cell lines (Figure 1M–O; Figure S2C, D).

**FIGURE 2:**
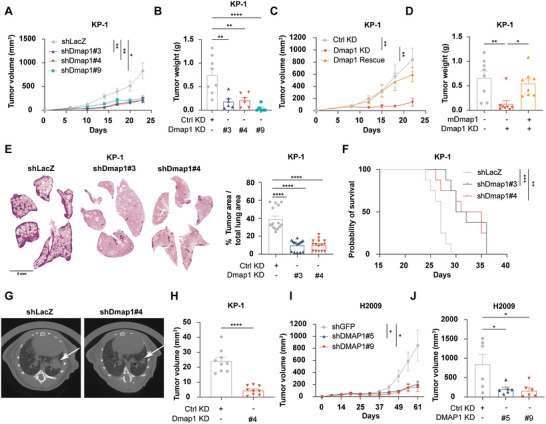
Dmap1 knockdown attenuates lung cancer progression in vivo. (A) Tumor growth curve of KP‐1‐shLacZ and KP‐1‐shDmap1 in C57BL/6 subcutaneous model, shLacZ, *n* = 8; shDmap1#3, shDmap1#4, shDmap1#9, *n* = 6. (B) Endpoint tumor weight of (A). (C) Tumor growth curve of KP‐1 Dmap1 rescue in C57BL/6 allograft model, *n* = 8. (D) Endpoint tumor weight of (C). (E) Representative fields (left) and quantification (right) of lung tumor burden for C57BL/6 KP‐1 i.v. model, *n* = 15. (F) Kaplan‐Meier survival curve of C57BL/6 KP‐1 i.v. model, *n* = 8. (G), Representative CT sections for shLacZ and shDmap1 in C57BL/6 KP‐1 in situ allograft model. (H) Statistical analysis for (G), *n* = 9. (I) Tumor growth curve of H2009‐shGFP or H2009‐shDMAP1 in BALB/c nude mice subcutaneous model, shGFP, *n* = 8; shDmap1#5, shDMAP1#9, *n* = 6. (J) Endpoint tumor volume of (I). Data are presented as mean ± SEM. Data were analyzed using *t*‐test. ^*^, *p* < 0.05; ^**^, *p* < 0.01; ^***^, *p* < 0.001; ^****^, *p* < 0.0001.

We next employed C57BL/6 mouse allograft models to assess the impact of Dmap1 depletion on lung tumor progression in vivo. In the subcutaneous model, Dmap1 knockdown significantly suppressed KP‐1 tumor progression, while re‐expression of Dmap1 fully restored tumor growth (Figure 2A–D; Figure S3A, B). Immunohistochemical staining revealed reduced Ki67 expression in Dmap1‐KD tumors, indicating decreased proliferation (Figure S3C, D). Similarly, in the tail vein intravenous (i.v.) injection model, Dmap1 knockdown reduced lung tumor burden and extended mouse survival (Figure 2E, F). To better mimic the native tumor microenvironment, we further utilized in situ lung orthotopic transplantation tumor models followed by CT scanning, and tumor volumes were markedly lower in the Dmap1‐KD group (Figure 2G, H). Consistently, Dmap1 knockdown in human LUAD H2009 cells impaired subcutaneous tumor growth in BALB/c nude mice (Figure 2I, J; Figure S3E). These findings support a critical role for DMAP1 in promoting lung tumorigenesis in both murine and human models.

### 2.3 Dmap1 Knockdown Triggers Cell Cycle Abnormality

**FIGURE 3:**
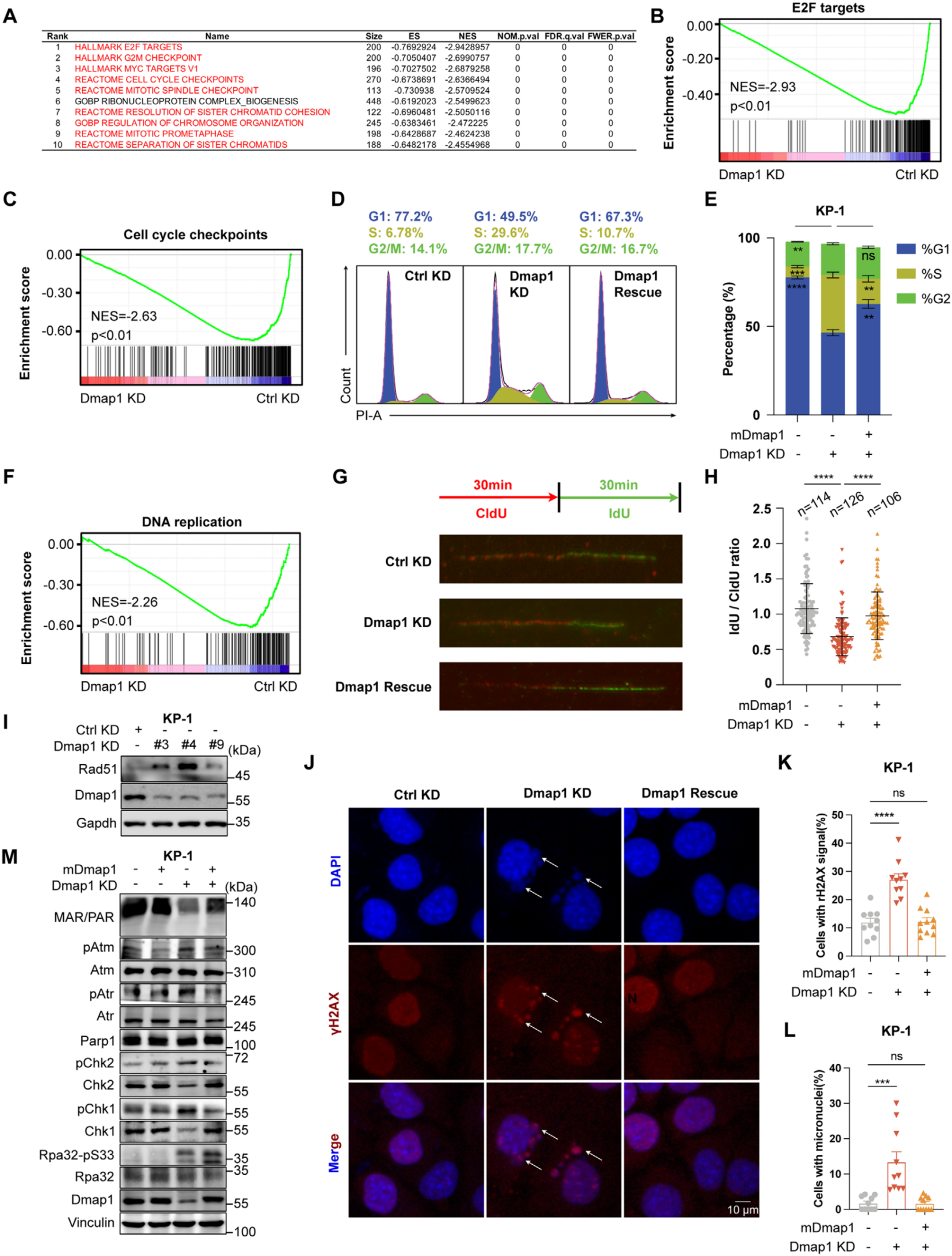
Dmap1 knockdown triggers cell cycle abnormality and DNA damage. (A) GSEA showing the top 10 enriched pathways in KP‐1 cells with Dmap1 knockdown. (B) GSEA showing downregulation of genes associated with “E2F_TARGETS” in KP‐1 cells with Dmap1 knockdown. (C) GSEA showing downregulation of genes associated with “CELL_CYCLE_CHECKPOINTS” in KP‐1 cells with Dmap1 knockdown. (D) Representative flow cytometry analysis of cell cycle distribution across all KP‐1 Dmap1 rescue groups. (E) Statistical analysis for (D), *n* = 3. (F) GSEA showing downregulation of genes associated with “DNA_REPLICATION” in KP‐1 cells with Dmap1 knockdown. (G) Schematic for labeling cells with CIdU (Red) and IdU (Green) and representative fields of DNA fibers across all KP‐1 Dmap1 rescue groups. (H) Statistical analysis of the ratio between CldU and IdU for (G). (I) Dmap1 knockdown increases Rad51 levels in KP‐1 cells. (J) Micronuclei and γ‐H2AX foci accumulation in Dmap1‐deficient KP‐1 cells detected by IF. (K) Statistical analysis of γ‐H2AX foci for (J), Ctrl KD, *n* = 10, Dmap1 KD, *n* = 10, Dmap1 rescue, *n* = 11. Each data point represents a blind field. (L) Statistical analysis of micronuclei for (J), Ctrl KD, *n* = 10, Dmap1 KD, *n* = 10, Dmap1 rescue, *n* = 11. Each data point represents a blind field. (M) KP‐1 cells with Dmap1 WT, OE, KD, and rescue cells were analyzed using DNA‐damage‐related antibodies. Data are presented as mean ± SEM. Data were analyzed using *t*‐test. ns, not significant; ^**^, *p* < 0.01; ^***^, *p* < 0.001; ^****^, *p* < 0.0001.

Given the impaired colony formation observed in the Dmap1‐KD cells, we first assessed apoptosis using Annexin V/7‐AAD flow cytometry. However, Dmap1 knockdown did not enhance apoptosis in either KP‐1 or H2009 cells (Figure S4A–C). To further explore the mechanistic role of Dmap1 in lung cancer progression, we performed bulk RNA sequencing in KP‐1 cells to identify differentially expressed genes and pathways upon Dmap1 knockdown. Gene set enrichment analysis (GSEA) of RNA‐seq data revealed significant depletion of cell cycle‐related pathways in Dmap1‐KD cells (Figure 3A–C). Therefore, we next evaluated cell cycle dynamics via PI staining, which showed an increased proportion of S‐phase cells and reduced G1‐phase cells, with only minor change in the G2/M‐phase population (Figure 3D, E), which suggested a possible cell cycle abnormality and DNA replication blockade, leading to an increase in the number of cells with DNA content between 2 and 4N.

To precisely pinpoint the nature of this observed cell cycle abnormality, we further analyzed the physical parameters of the cells derived from the flow cytometry data. Quantitative analysis revealed a statistically significant elevation in both forward scatter (FSC) and side scatter (SSC) in Dmap1‐KD cells (Figure S4D, E), indicating a profound increase in overall cell size and intracellular granularity, respectively. To morphologically validate these physical shifts, we performed immunofluorescence (IF) co‐staining of β‐actin and DAPI to delineate the cytoplasmic and nuclear boundaries. Confocal microscopy imaging strikingly corroborated the flow cytometric findings: Dmap1‐KD cells exhibited a massive expansion in overall cytoplasmic volume, characteristic of cellular hypertrophy (Figure S4F). More importantly, we observed the emergence of cells possessing abnormally enlarged nuclei, or karyomegaly (Figure S4F, G). Collectively, these robust quantitative and morphological readouts explicitly explain the observed cell cycle defect.

Since CDKs and Cyclins are critical regulators of the cell cycle, we examined their changes following Dmap1 perturbation. Dmap1 knockdown significantly upregulated Cyclin D1 and Cdk4/6 but downregulated Cyclin E2 and Cdk2, with no significant change in Cyclin A2 levels (Figure S4H). To further assess the functional activation of the CDK kinases, we examined their specific phosphorylation status. While the levels of pCdk1‐T160/pCdk2/3‐T161, pCdk1/2/3‐T14, and pCdk4‐T172 remained largely unaltered, we observed a striking reduction in the inhibitory phosphorylation of Cdk6 at Thr24 (Figure S4D). Cyclin D1 and Cdk4/6, which are active in G1 phase, promote G1/S transition by phosphorylating Rb to release E2F transcription factors [21], while Cyclin E2 and Cdk2 are upregulated at the G1‐to‐S transition to initiate DNA replication [22]. Our observation of the S‐phase abnormality following DMAP1 knockdown (Figure 3D, E), together with the downregulation of Cyclin E2 and Cdk2, suggests the operation of a feedback inhibitory mechanism. Concurrently, the decrease in inhibitory phosphorylation, coupled with the increased abundance of the Cyclin D1/Cdk6 complex, strongly suggests a compensatory activation of Cdk6 kinase in response to the replication stress, which represents an unsuccessful attempt to drive cell cycle progression. Collectively, these findings indicate that Dmap1 knockdown suppresses lung cancer proliferation primarily by inducing cell cycle abnormality.

### 2.4 Dmap1 Knockdown Causes Replication Fork Retardance and DNA Damage

DMAP1 was reported to localize at the replication fork during S‐phase, contributing to DNA methylation maintenance and transcriptional silencing on newly synthesized DNA strands [14, 23]. To further investigate its potential role in replication, we performed IP‐MS experiments to profile Dmap1‐interacting proteins. STRING annotation and clustering analysis of the results identified a network of Dmap1‐interacting proteins (Figure S4I), including members of the Tip60 complex (Ep400, Trrap, Actl6a, Brd8, et al.) and histone H2A.Z. This finding is consistent with prior studies identifying Dmap1 as a component of the Tip60 complex [24] and validates the reliability of our IP‐MS data.

Notably, our IP‐MS screen revealed multiple DMAP1 interactors closely linked to replication fork dynamics. These included the core replication‐initiation complex (Mcm2, Mcm4, Mcm5) responsible for DNA unwinding and replication initiation (Figure S4I, J), the FACT complex (Ssrp1, Supt16) that ensures smooth replication‐fork progression, members of the cohesin complex (Smc1a), and the MRN complex (Mre11a), which are both involved in protecting and repairing stalled replication forks. These interactions further support Dmap1's role in replication fork regulation.

Prompted by these protein interactions and the observed downregulation of DNA replication pathways in the Dmap1‐KD cells (Figure 3F), we directly assessed replication fork dynamics using a DNA fiber assay. Dmap1‐KD cells showed a significant decrease in fork symmetry, as evidenced by a decrease in the IdU (green) to CldU (red) ratio, indicating the occurrence of replication fork stalling (Figure 3G, H). To further interpret the results, we quantitatively analyzed the absolute length of CldU (red) tracks to better characterize the initial fork elongation dynamics. Interestingly, the CldU track lengths are significantly increased in the Dmap1‐KD cells compared to control cells (Figure S4K). Although seemingly paradoxical, the dual phenotypes—initial elongation followed by subsequent stalling—align with the emerging paradigm of “accelerated but unstable fork progression” as a profound source of replication stress [25]. In line with these results, Rad51, a key replication fork stabilizer [26], was notably upregulated in Dmap1‐KD cells (Figure 3I), suggesting an activated compensatory response to replication stress. Furthermore, the iPOND assay confirmed that Dmap1 can localize at the replication fork, suggesting its involvement in replication fork function (Figure S4L). Crucially, this profound replication crisis at the molecular level provides a direct mechanistic basis for the macroscopic cellular phenotypes we characterized above. Driven by the severe DNA replication blockade, these cells halt their division to prevent mitotic entry with compromised genomes, while continuing macromolecular synthesis, ultimately culminating in the observed cellular hypertrophy and nuclear enlargement (Figure S4D–G).

Replication fork stalling is a known driver of genomic instability and DNA damage, which generates single‐strand DNA (ssDNA) that activates ATR‐CHK1 signaling and the intra‐S checkpoint, whereas replication fork collapse produces double‐strand breaks that trigger ATM‐CHK2 activation [27, 28, 29, 30], which prompted us to examine DNA damage status in Dmap1‐KD cells. The alkaline comet assay shows that Dmap1 knockdown induces DNA damage in KP‐1 cells (Figure S4M, O). It is well‐established that unresolved DNA replication stress leads to the missegregation of acentric chromosome fragments during mitosis, culminating in the formation of micronuclei. IF staining showed Dmap1 knockdown increased the formation of micronuclei and γ‐H2AX foci in and around the nuclei of Dmap1‐KD KP‐1 cells (Figure 3J–L). Notably, we also observed a strong colocalization of robust γ‐H2AX signals within these micronuclei following Dmap1 knockdown (Figure 3J–L). This dense accumulation of γ‐H2AX not only reflects the primary unresolved DNA lesions partitioned into the micronuclei, but also signifies the catastrophic secondary DNA damage typically resulting from the spontaneous collapse of the fragile micronuclei envelope, a hallmark of genomic instability [31, 32].

To further determine the type of DNA damage induced by Dmap1 knockdown, we further interrogated DNA damage‐related markers (Figure 3M). Dmap1 knockdown upregulated both the Atr‐Chk1 and Atm‐Chk2 signaling, suggesting the occurrence of both ssDNA exposure and dsDNA breaks. While Chk1‐pS345 detects ssDNA exposure caused by replication stalling, RPA‐pS33 is upregulated when ssDNA breaks lead to replication collapse. We also assessed whether Dmap1 knockdown induces DNA damage via oxidative stress using an 8‐oxo‐dG Elisa assay, but no significant differences were observed (Figure S4P), and MAR/PARylation level, which is mainly induced by ROS stress, Atr‐Chk1 inhibition, or alkylation agents, didn't increase (Figure 3M). Together, these findings suggest that Dmap1 knockdown induces ssDNA exposure and ssDNA breaks, which leads to replication fork collapse, and ultimately leads to more severe dsDNA damage and micronuclei formation.

### 2.5 Dmap1 Knockdown Boosts Anti‐tumor Immunity

**FIGURE 4:**
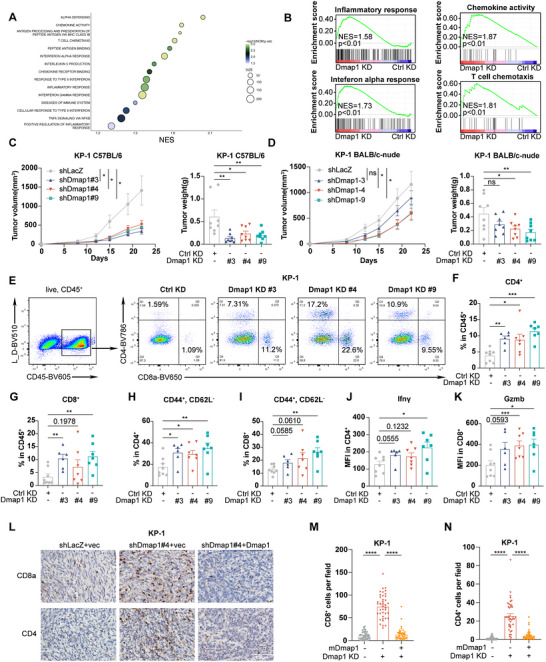
Dmap1 knockdown boosts anti‐tumor immunity. (A) Bubble plot showing the enrichment of immune‐related pathways in KP‐1 Dmap1 knockdown cells. (B) GSEA showing enrichment of genes associated with “INFLAMMATORY_RESPONSE”, “INTERFERON_ALPHA_RESPONSE”, “CHEMOKINE_ACTIVITY”, and “T_CELL_CHEMOTAXIS” in KP‐1 cells with Dmap1 knockdown. (C) Tumor growth curve (left) and endpoint tumor weight (right) of KP‐1‐shLacZ or KP‐1‐shDmap1 in C57BL/6 subcutaneous model, *n* = 8. (D) Tumor growth curve (left) and endpoint tumor weight (right) of KP‐1‐shLacZ or KP‐1‐shDmap1 in BALB/c‐nude mice subcutaneous model, *n* = 8. (E) Representative flow analysis of CD4^+^ and CD8^+^ populations in CD45^+^ immune cells. (F), (G) Flow analysis of CD4^+^ (F), CD8^+^ (G) T cells. (H), (I) Flow analysis of CD44^+^, CD62L^−^ in CD4^+^ (H) and CD8^+^ (I) T cells. (J) Flow analysis of Ifnγ MFI in CD4^+^ populations. (K) Flow analysis of Gzmb MFI in CD8^+^ populations. (F–K), *n* = 8 (Ctrl KD), 6 (shDmap1#3), 7 (shDmap1#4), and 7 (shDmap1#9). (L) Representative fields of IHC staining in KP‐1 C57BL/6 subcutaneous tumor samples stained for CD8a and CD4. (M), (N), Statistical analysis for (L), *n* = 40. Each data point represents a blind field. Data are presented as mean ± SEM. Data were analyzed using *t*‐test. ns, not significant; ^*^, *p* < 0.05; ^**^, *p* < 0.01; ^***^, *p* < 0.001; ^****^, *p* < 0.0001.

In our bulk RNA‐seq data, immune‐related pathways, including INFLAMMATORY_RESPONSE and INTERFERON_ALPHA_RESPONSE, were significantly upregulated in Dmap1‐KD cells (Figure 4A, B). Among these immune‐regulating genes, IFN‐stimulated genes (ISGs) showed the most pronounced changes by Dmap1 loss (Figure S5). To further determine whether Dmap1 knockdown induces anticancer immune activation in vivo, we conducted parallel allograft experiments using both immunocompetent C57BL/6 and immunodeficient BALB/c nude mice. We inoculated Ctrl‐KD and Dmap1‐KD KP‐1 cells subcutaneously into those mice at the same time and monitored tumor growth. While Dmap1 knockdown suppressed tumor progression in both models, the effect was markedly enhanced in immunocompetent mice (Figure 4C, D), indicating that Dmap1 knockdown elicits an immune‐mediated anti‐tumor response.

To elucidate how Dmap1 deficiency enhances anti‐tumor immunity, we analyzed the immune profile of subcutaneous tumors by flow cytometry (Figure S6A). Although the overall abundance of CD45^+^ immune cells remained unchanged (Figure S6B), Dmap1 knockdown significantly increased infiltration of both CD4^+^ and CD8^+^ T cells (Figure 4E–G). Furthermore, there was a shift toward effector memory CD4^+^ and CD8^+^ T cell phenotypes, with elevated levels of CD44^+^CD62L^−^ effector T cells and reduced CD44^−^CD62L^+^ naïve T cells (Figure 4H, I; Figure S6C, D). Notably, IFNγ^+^ T cells and Gzmb^+^ cytotoxic CD8^+^ T cells were markedly enriched in Dmap1‐KD tumors (Figure 4J, K; Figure S6E), indicating enhanced T cell activation and cytotoxic potential. Furthermore, Ly6G^+^ polymorphonuclear myeloid‐derived suppressor cells (PMN‐MDSC), known for their immunosuppressive roles, were markedly reduced in Dmap1‐KD tumors (Figure S6F). However, no significant changes were observed in other immune cell subtypes (Figure S6G–K).

To more intuitively investigate T cell activation status, we performed multiplex immunofluorescence co‐staining of CD8a/CD69, CD4/CD69, and CD4/T‐bet on the FFPE KP‐1 subcutaneous tumors. Compared with WT tumors, Dmap1 knockdown tumors showed a significant increase in CD69^+^CD8^+^ cells, CD69^+^CD4^+^ cells, and the overall proportion of CD69‐positive T cells, indicating a more robust activation state of T cells (Figure S7A–F). Moreover, an increased proportion of T‐bet^+^ CD4 T cells was also observed, which indicated a Th1‐driven, inflamed, and anti‐tumor immune response, representing a more effective anti‐tumor immunity in Dmap1‐KD tumors (Figure S7G–I). IHC staining further confirmed increased infiltration of CD4^+^ and CD8^+^ T cells in these tumors (Figure 4L–N). Together, these results demonstrate that Dmap1 deficiency promotes an immunostimulatory tumor microenvironment by enhancing T cell infiltration and activity.

### 2.6 Dmap1 Deficiency Stimulates Type I Interferon Response through cGas‐Sting Activation

**FIGURE 5:**
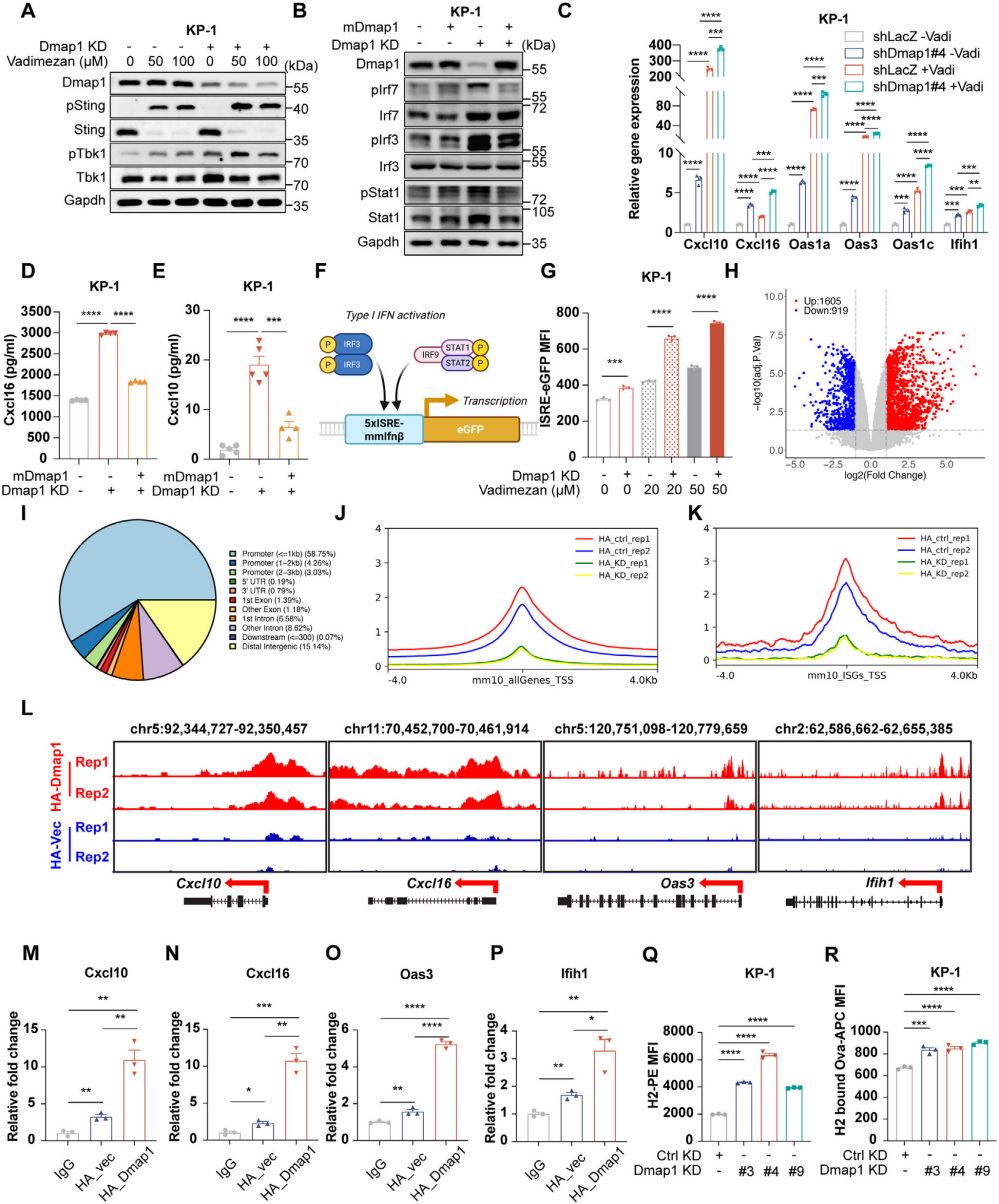
Dmap1 knockdown activates tumor cell‐intrinsic immune signaling through type I IFN signaling. (A) Dmap1 knockdown increases pTbk1, total Tbk1, and pSting levels in KP‐1 cells treated with 50 µм or 100 µм Vadimezan for 4 h. (B) Dmap1 knockdown increases pStat1, total Stat1, pIrf3, pIrf7, and total Irf7 levels in KP‐1 cells. (C) qRT‐PCR shows that Dmap1 knockdown upregulates ISGs expression at the mRNA level in baseline or 50 µм, 4 h Vadimezan‐treated KP‐1 cells, *n* = 3. (D), (E) Elisa assay shows Dmap1 knockdown upregulates Cxcl16 (D, *n* = 4) and Cxcl10 (E, *n* = 5, 4, 4) at the protein level. (F) Schema of the ISRE binding site‐based type‐I signaling reporter. Created in BioRender. Huang, K. (2026) https://BioRender.com/ruziswb (G) ISRE‐eGFP signal of shLacZ and shDmap1 KP‐1 cells. The indicated cells were transfected with ISRE‐eGFP reporter, treated with 0, 20, or 50 µм Vadimezan for 4 h, and the eGFP signals were detected using flow cytometry, *n* = 3. (H) Volcano plot of DEGs between Dmap1‐KD and Ctrl‐KD KP‐1 cells (3 duplicates, adjusted *p* < 0.05, |log_2_foldchange| > 1). (I) Genomic distribution of Dmap1 peaks in KP‐1 cells. (J) The binding pattern for Dmap1 at the promoter regions of mouse genes. (K) The binding pattern for Dmap1 at the promoter regions for mouse IFN signaling genes. (L) Genome browser tracks of Dmap1 CUT&Tag at genomic loci of *Cxcl10*, *Cxcl16*, *Oas3*, and *Ifih1*. (M–P) CUT&Tag‐PCR for Dmap1's binding on the promoter region of *Cxcl10* (M), *Cxcl16* (N), *Oas3* (O), and *Ifih1* (P). (Q) Flow analysis shows Dmap1 knockdown upregulates H2 presentation in KP‐1 cells. The indicated KP‐1 cells were stimulated with 20 ng/mL mouse IFNγ for 48 h before harvest, *n* = 3. (R) Flow analysis shows Dmap1 knockdown upregulates H2‐bound Ova presentation in KP‐1 cells. The indicated KP‐1 cells were transfected with Ova peptide and stimulated with 20 ng/mL mouse IFNγ for 48 h before harvest, *n* = 3. Data are presented as mean ± SEM. Data were analyzed using *t*‐test. ^*^, *p* < 0.05; ^**^, *p* < 0.01; ^***^, *p* < 0.001; ^****^, *p* < 0.0001.

Micronuclei formation caused by DNA damage has recently been implicated in the activation of immune responses, primarily through the cGas‐Sting pathway, which senses cytosolic DNA and induces type I IFN signaling [33, 34, 35]. Therefore, we further explored whether the depletion of Dmap1 could enhance the cGas‐Sting activity. The baseline phosphorylation amounts of endogenous Tbk1 were higher in Dmap1 knockdown cells, and Dmap1 deficiency strengthened the effects of Sting agonist Vadimezan (Figure 5A), demonstrating the activation of cGas‐Sting signaling upon Dmap1 deficiency. To further investigate the change of downstream signaling of cGas‐Sting upon Dmap1 knockdown, we used western blot analysis and confirmed that Dmap1 depletion activated type I IFN signaling, as indicated by the elevated levels of pIrf3, pIrf7, and pStat1 (Figure 5B). Consistent with our RNA‐seq data (Figure S5), qRT‐PCR results showed that the expression of interferon‐stimulated genes (ISGs) was increased in the Dmap1 knockdown group, and Dmap1 deficiency strengthened the effects of Vadimezan (Figure 5C). Notably, Cxcl16 and Cxcl10, chemokines involved in cytotoxic T cell recruitment, were upregulated at both mRNA and protein levels (Figure 5D, E). To further assess IFN activation, we used an interferon‐sensitive response element (ISRE)‐based reporter (Figure 5F), and flow cytometry analysis showed that Dmap1‐KD cells displayed increased eGFP signal, confirming cell‐intrinsic activation of type I IFN signaling (Figure 5G). Consistently, in the TCGA LUAD and LUSC cohorts, DMAP1 expression showed a negative correlation with key T cell‐recruiting chemokines, including *CXCL9*, *CXCL10*, *CXCL11*, and *CCL5* (Figure S7J), further supporting its role in immune modulation.

Previous studies have reported that phagocytosis of DNA‐damaged cells or tumor‐derived DNA can induce cGAS‐STING signaling, thus activating antigen‐presenting cells [36, 37]. To determine whether a similar process occurs in our system, we performed multiplex immunofluorescence staining on KP‐1 subcutaneous tumor tissues with or without Dmap1 knockdown, analyzing the positivity and colocalization of dsDNA and the macrophage marker F4/80. Compared to WT controls, Dmap1 knockdown tumors exhibited a marked increase in dsDNA‐positive cells, corroborating our finding that Dmap1 loss promotes dsDNA accumulation (Figure S7K, L). Notably, we also observed a significant increase in the number of F4/80 and dsDNA double‐positive cells, suggesting that in Dmap1‐KD tumors, macrophages may engulf tumor‐derived dsDNA via phagocytosis (Figure S7K, M). These findings imply a potential role for macrophages in mediating anti‐tumor immunity in our context.

To summarize, these findings highlight the fact that Dmap1 knockdown activates cell‐intrinsic type I IFN signaling and promotes T cell‐recruiting chemokine expression through cGas‐Sting stimulation.

### 2.7 Dmap1 Deficiency Additionally Up‐regulates ISGs through Transcriptional De‐repression

Previous studies have reported that DMAP1 functions as a transcriptional repressor by interacting with epigenetic silencing factors [14, 38]. Therefore, we wanted to examine whether Dmap1 could directly suppress the transcription of ISGs, independent of cGas‐Sting signaling. Analysis of RNA‐seq data revealed that the majority of differentially expressed genes (DEGs) were significantly upregulated upon Dmap1 knockdown, suggesting a repressor role for Dmap1 (Figure 5H). To further validate this, we performed CUT&Tag profiling in KP‐1 cells using HA‐tagged Dmap1 (Figure S8A). As expected, Dmap1‐KD KP‐1 cells exhibited a markedly reduced number of Dmap1‐binding sites compared to WT cells (Figure S8B). Notably, over half of these binding sites were located within promoter regions (Figure 5I), and Dmap1 depletion significantly diminished promoter occupancy (Figure 5J). Integrative analysis of RNA‐seq and CUT&Tag data revealed that 1,201 genes were potential targets of Dmap1 (Figure S8C). Gene ontology analysis of those genes showed enrichment of immune‐related pathways, including T cell‐mediated cytotoxicity, antigen‐presenting, and inflammatory response (Figure S8D). More importantly, Dmap1 was found to bind to the promoter region of a group of IFN signaling genes common to both mouse and human [39], including several canonical ISGs, such as Cxcl10, Cxcl16, Oas3, and Ifih1 (Figure 5K, L), implicating it as a direct transcriptional repressor of type I IFN signaling. We further quantified the binding of Dmap1 on the promoter region of differentially expressed ISGs, including Cxcl10, Cxcl16, Oas3, and Ifih1, using CUT&Tag‐qPCR. Compared with IgG and Dmap1‐KD groups, Ctrl‐KD KP‐1 cells have significant amplification of Dmap1 binding on the intended ISGs (Figure 5M–P). Furthermore, analysis of public ChIP‐seq data from the human HepG2 cell line revealed pronounced DMAP1 binding peaks at the promoter regions of CXCL16, OAS3, and IFIH1 (Figure S8E), suggesting that DMAP1‐mediated regulation of ISG transcription may be a conserved mechanism. Motif analysis of Dmap1 peaks in 1,201 DEGs using HOMER revealed that Fos and JunB motifs were the top hits among the results, which were known inflammation regulators, implicating that Dmap1 might regulate inflammation through AP‐1 family members (Figure S8F). Collectively, these findings suggest that, in addition to cGas‐Sting activation, Dmap1 restrains interferon responses by directly suppressing ISG transcription at the promoter level.

### 2.8 Dmap1 Deficiency Strengthens Antigen Presentation and Immune‐checkpoint Expression

Cell‐intrinsic activation of type I IFN signaling is known to enhance antigen presentation [13]. Consistently, Dmap1 knockdown markedly upregulated MHC‐I expression and antigen presentation capacity in vitro (Figure 5Q, R). The elevated MHC‐I expression induced by Dmap1 knockdown was also observed in the in vivo immune profiling data (Figure S8G). These results suggest that Dmap1 deficiency may promote T cell activation by enhancing tumor immunogenicity. Additionally, PD‐L1 expression was elevated in Dmap1‐KD tumors (Figure S8H), and DMAP1 expression was negatively correlated with immune checkpoint molecules such as *CD274*, *HAVCR2*, and *PDCD1LG2* in the TCGA LUAD dataset (Figure S8I). Overall, these results underscore the importance of DMAP1 perturbation in promoting tumor immunogenicity and optimizing the response to immunotherapy.

### 2.9 Single‐cell Analysis of Tumor Microenvironment Confirms that Knockdown of Dmap1 Potentiates T‐cell Activation

**FIGURE 6:**
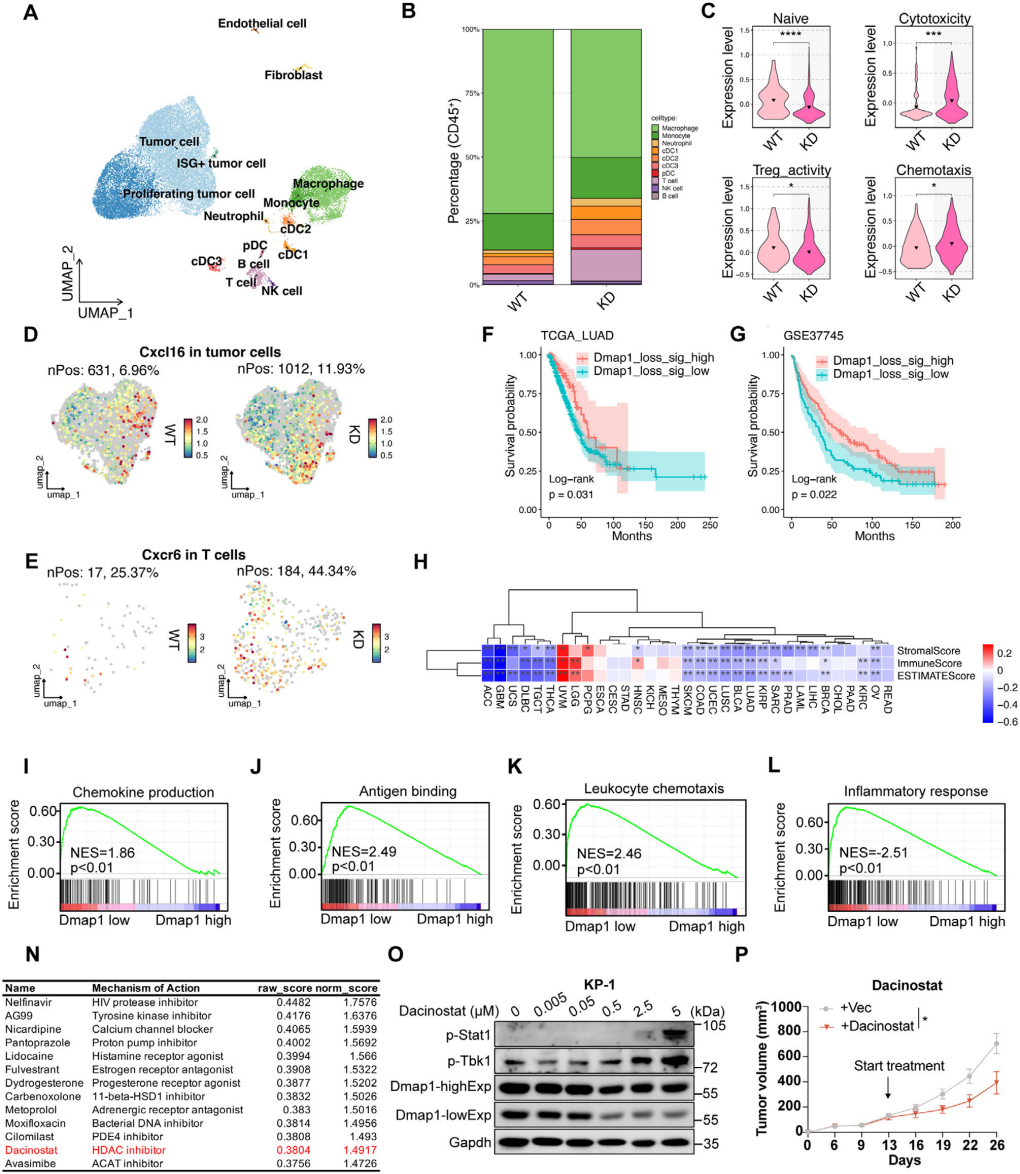
Dmap1 perturbation potentiates T cell activation and infiltration and correlates with worse clinical outcomes. (A) UMAP plot showing clusters of tumor cells and intratumoral immune cell populations. (B) Changes in the different immune compartments in response to Dmap1 knockdown. (C) Changes in inferred signature expression level in T cells in response to Dmap1 knockdown, the inverted triangle represents the median level. (D) UMAP plot showing changes in Cxcl16 expression level in tumor cells in response to Dmap1 knockdown. (E) UMAP plot showing changes in Cxcr6 expression level in T cells in response to Dmap1 knockdown. (F‐G) Kaplan‐Meier survival curves for patients with DMAP1‐loss signature high‐ versus low‐expressing tumors in the TCGA LUAD (F) and GSE37745 (G) cohorts. (H), The heatmap shows the Spearman correlation of DMAP1 expression versus ESTIMATE score in various TCGA cohorts. (I–L), Enrichment of genes associated with “CHEMOKINE_PRODUCTION”, “ANTIGEN_BINDING”, “LEUKOCYTE_CHEMOTAXIS”, and “INFLAMMATORY_RESPONSE” in patients with low DMAP1‐expressing tumors. (M), L1000 predicted the top 13 drugs that had either undergone clinical trials or received FDA approval. (N), WB showed Dacinostat inhibited Dmap1 expression and interferon pathway activation; cells were treated for 24 h before harvested. (O) Tumor growth curve of KP‐1 tumors with Dacinostat treatment in C57BL/6 subcutaneous allograft model, *n* = 14. Data were analyzed using Wilcoxon rank‐sum test [(C)] and *t*‐test [(O)]. ^*^, *p* < 0.05; ^**^, *p* < 0.01; ^***^, *p* < 0.001; ^****^, *p* < 0.0001.

To provide a comprehensive assessment of the tumor microenvironment affected by Dmap1 deficiency, we performed single‐cell RNA‐seq (scRNA‐seq) on C57BL/6 subcutaneous KP‐1 tumors. We identified distinct tumor cell, macrophage, monocyte, neutrophil, DC, T cell, B cell, and NK cell clusters (Figure 6A; Figure S9A, B). Consistent with our previous observations, there was a remarkable increase in the T cell cluster in the Dmap1‐KD (Figure 6B; Figure S9A–C). Interestingly, the three classical DC (cDC) clusters with high *Zbtb46* expression also showed a concurrent increase in the Dmap1‐KD tumors (Figure 6A, B). Together with the increased abundance and activation of T cells, this suggests a highly immunogenic microenvironment in Dmap1‐KD tumors.

To further evaluate how Dmap1 deficiency affected the T cell transcriptome, we performed gene signature score analysis for these cells. Compared to WT tumors, T cells in the Dmap1‐KD tumors exhibited reduced naïve T cell phenotype and elevated cytotoxicity, which aligned with our immune profiling results (Figure 6C). Furthermore, T cells in the Dmap1‐KD group showed less regulatory activity, whilst expressing higher levels of chemokine and chemokine receptors (Figure 6C). These findings indicate that Dmap1 knockdown enhances both T cell recruitment and subsequent activation.

To further interrogate the cytokine–receptor pairs by which tumor cells promote T cell infiltration, we analyzed the expression patterns of classical T cell chemoattractants and their corresponding receptors in tumor and T cells based on single‐cell transcriptomic data. Consistent with our RT‐qPCR and Elisa results (Figure 5C,D), Cxcl16, as an ISG, which was reported to be critical for sustained tumor control mediated by CD8^+^ cytotoxic T cells, was highly expressed by Dmap1‐KD tumor cells (Figure 6D). Its receptor Cxcr6 was also enriched in Dmap1‐KD tumor‐infiltrating T cells (Figure 6E). Consistent with our previous observations, we also found that mouse MHC‐I molecules H2‐K1 and H2‐D1 were highly expressed in the Dmap1‐KD tumor cells (Figure S9D). CellChat analysis also revealed that in the Dmap1‐KD group tumors, interactions between T cells and other cells were significantly more abundant (Figure S9E). Collectively, Dmap1‐KD tumor cells upregulated antigen presentation and Cxcl16 expression, and potentially promote Cxcr6^+^ T cell infiltration, suggesting that the Cxcl16‐Cxcr6 axis may serve as a key pathway mediating enhanced anti‐tumor immunity in this context.

### 2.10 DMAP1 Correlates with a Cold Immune Microenvironment and Worse Clinical Outcomes

To further investigate the clinical significance of DMAP1, we constructed a DMAP1‐loss signature using the homolog of the top 100 up‐regulated genes in Dmap1‐KD KP‐1 cells. In the TCGA LUAD cohort and NSCLC cohort GSE37745 [40], a higher DMAP1‐loss signature was associated with improved overall survival (OS) (Figure 6F, G). Using the ESTIMATE algorithm [41], we found that DMAP1 negatively correlated with immune cell infiltration and contributed to a “cold” tumor microenvironment in most TCGA cancer types, including LUAD and LUSC, which was further validated in the GSE37745 lung cancer cohort (Figure 6H; Figure S9F). Similarly, anti‐tumor immunity‐related pathways were up‐regulated in DMAP1‐low samples compared to DMAP1‐high samples in the TCGA LUAD cohort (Figure 6I‐L), suggesting that DMAP1 expression levels correlate with a less immune‐activated tumor microenvironment and poorer patient prognosis.

### 2.11 Dacinostat was Identified as a Dmap1 Inhibitor with In Vivo Immune‐mediated Anti‐tumor Effects

Given that Dmap1 functions as a potential anti‐tumor therapeutic vulnerability, we sought to pharmacologically mimic Dmap1 knockdown to recapitulate its anti‐tumor immune effects. However, there are currently no available Dmap1 inhibitors. Therefore, we used the L1000 platform to predict potential Dmap1 inhibitors based on the transcriptional changes observed in KP‐1 cells following Dmap1 knockdown [42]. The top 150 up‐regulated and down‐regulated differentially expressed genes from our RNA‐seq data were used for L1000 prediction, and the top 13 compounds that had either undergone clinical trials or received FDA approval were selected as candidates for further experimental validation (Figure 6M). 12 of these compounds did not exhibit any ability to suppress Dmap1 expression (Figure S10A). Interestingly, Dacinostat, an HDAC inhibitor, was able to suppress Dmap1 expression in a dose‐dependent manner and activate the downstream expression of pTbk1 and pStat1 (Figure 6N).

Furthermore, we performed therapeutic studies using the KP‐1 subcutaneous tumor model. KP‐1 cells were inoculated subcutaneously into immunocompetent C57BL/6 mice, and Dacinostat treatment was initiated when the tumor volume reached around 100 mm^3^. Mice in the treatment group received daily intraperitoneal injections of Dacinostat at the concentration of 5 mg/kg, while control mice were administered with the vehicle. Notably, Dacinostat treatment markedly reduced tumor volume in KP‐1‐bearing mice (Figure 6O). Therefore, Dacinostat significantly suppressed tumor progression in vivo. Flow cytometry analysis of the immune microenvironment revealed that Dacinostat promoted the infiltration of both CD4^+^ and CD8^+^ T cells, a phenotype consistent with that observed upon Dmap1 knockdown (Figure S10B). These results indicated that Dacinostat, as a Dmap1 inhibitor, exerts anti‐tumor immune effects and highlights its potential for future clinical applications.

**FIGURE 7:**
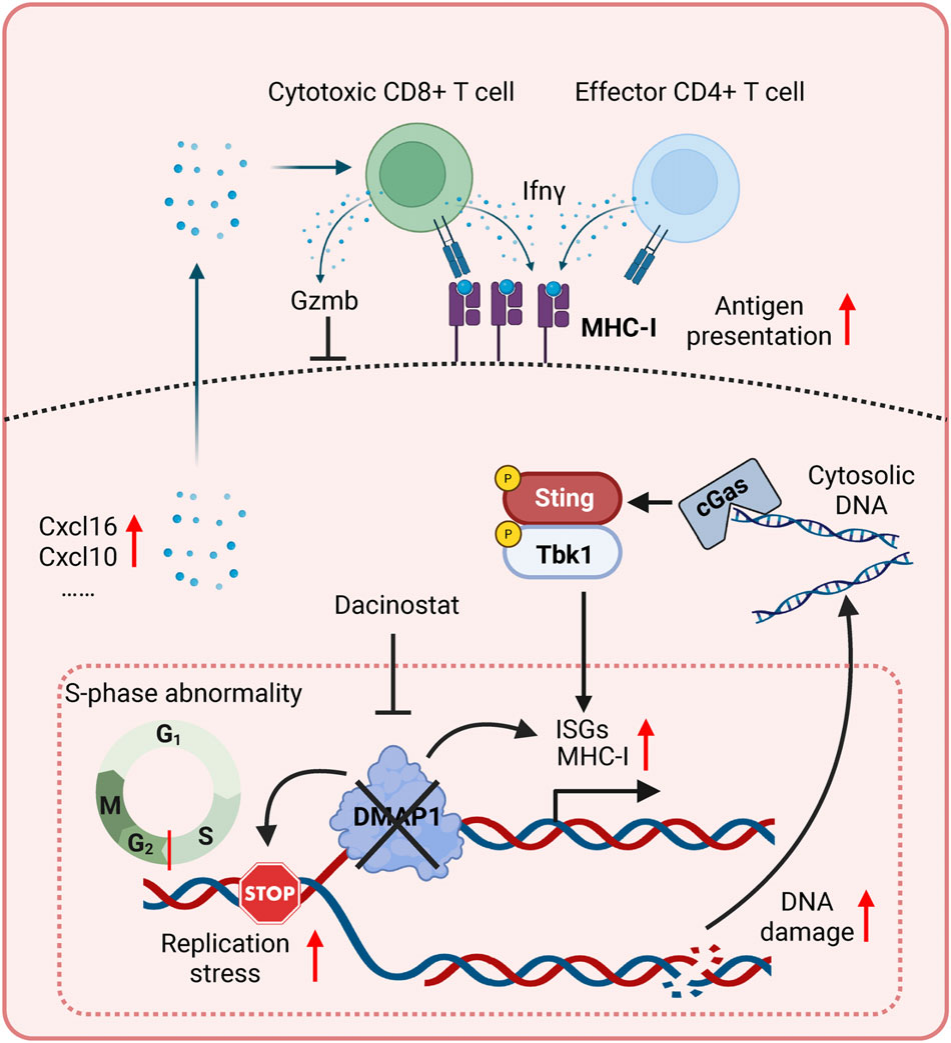
Working model for how Dmap1 deficiency hinders lung cancer progression. Tumor cell‐intrinsic Dmap1 deficiency causes S‐phase abnormality and DNA damage by inducing replication fork stalling, which promotes micronuclei‐derived cytosolic DNA generation and subsequent cGas‐Sting and IFN signaling activation. Dmap1 deficiency concomitantly activates ISG transcription through transcriptional derepression. Therefore, Dmap1 deficiency promotes T cell infiltration and activation and boosts anti‐tumor immunity. Created in BioRender. Huang, K. (2026) https://BioRender.com/rqgn96u.

Collectively, our research demonstrated that deficiency of DMAP1 in cancer cells induces DNA replication stress and activates anti‐tumor T cell immunity through cell‐intrinsic type I IFN signaling (Figure 7), thereby uncovering a previously unrecognized epigenetic‐immune axis in NSCLC and highlighting DMAP1 as a potential therapeutic vulnerability.

## 3 Discussion

In this study, we identified DMAP1 as a previously unrecognized epigenetic regulator that promotes lung cancer progression through dual mechanisms—preserving replication fork stability and repressing type I interferon signaling. An epigenetic CRISPR screen revealed that Dmap1 was critical for lung cancer cell viability. Functional assays demonstrated that Dmap1 depletion induced replication fork stalling, cell cycle abnormality, and DNA damage, highlighting its important role in maintaining genomic stability. In vivo, knockdown of Dmap1 led to robust activation of type I IFN signaling, accompanied by enhanced antigen presentation and increased infiltration and activation of CD4^+^ and CD8^+^ T cells, indicating heightened tumor immunogenicity. Mechanistically, we show that Dmap1 knockdown activated cGas‐Sting signaling and transcriptionally upregulated ISG expression, contributing to an immunostimulatory tumor microenvironment. Furthermore, we used transcriptomic data to predict Dacinostat as a potential inhibitor of Dmap1 and validated its anti‐tumor efficacy in mice. These findings advance our understanding of the interplay between DMAP1, genome maintenance, and immune evasion, suggesting that disrupting DMAP1 may represent a promising therapeutic strategy to concurrently inhibit tumor proliferation and enhance anti‐tumor immunity.

Although our study uncovered key roles of DMAP1 in replication stress and immune modulation in KP lung cancer models as an oncogene, its role in other cancer types or contexts remains controversial and incompletely understood. In gastric cancer, Wang et al. discovered that, as a direct downstream interacting partner of MDGA2, DMAP1 significantly suppressed gastric cancer progression by activating the p53/p21 signaling cascade [16]. Yamaguchi et al. found that in neuroblastoma and fibroblast models, DMAP1 was identified as a new candidate as a tumor suppressor, whose expression activated p53 in an MYCN‐related, ATM‐dependent manner, and upregulated p53‐downstream pro‐apoptotic Bcl‐2 family molecules, resulting in p53‐induced apoptotic death [15]. Our work, together with these previous findings, highlights that DMAP1 may exert fundamentally distinct even opposing roles across different cellular contexts and p53 backgrounds, functioning as either a tumor suppressor or an oncogenic driver depending on the background setting.

We showed that Dmap1 knockdown induced DNA damage responses (DDR) in the KP model, consistent with previous findings implicating DMAP1 in genome maintenance and DNA repair processes [43, 44, 45]. Given that p53 is a central regulator of DDR and tumorigenesis [46], and prior studies have demonstrated that the function of DMAP1 in gastric cancer, neuroblastoma, MEFs, and hematopoietic stem cells is p53‐dependent [15, 16, 45], we examined whether this dependency applies to lung cancer models. Interestingly, despite aberrant p53 status in both the KP model and human H2009 cells, DMAP1 knockdown still exerted strong tumor‐suppressive effects. Moreover, knockdown of DMAP1 in p53 WT but LKB1‐mutant A549 cells also reduced colony formation (Figure S2). These findings suggest that the oncogenic role of DMAP1 in lung cancer may not be dependent on p53 or LKB1 mutation status, and its downstream mechanisms may differ across distinct genetic contexts.

In addition to forming a complex with DNMT1 during the cell cycle S phase, DMAP1 has also been described to interact with the PRC1 complex component BMI1 to recruit DNMT1, establishing DNA methylation at target gene promoters [47]. Moreover, DMAP1 is a core component of the Tip60‐p400 histone acetyltransferase (HAT) complex and the ATP‐dependent chromatin remodeling complex Swr1/SRCAP [45, 48]. In our IP‐MS data, we also detected members of the Tip60 complex (Ep400, Trrap, Actl6a, Brd8, et al.) among the Dmap1‐interacting proteins (Figure S4I). These observations suggest that DMAP1 may exert regulatory functions in both DNA methylation and histone modification in a context‐dependent manner, highlighting the need to investigate its role not only in our models but also across other cancer types to better define its therapeutic potential. Taken together, these insights underscore DMAP1 as a multifaceted regulator at the interface of epigenetic modification and cancer biology.

In our work, we demonstrated that DMAP1 deficiency activated cell‐intrinsic immune signaling through causing DDR and consequent cGas‐Sting activation, thereby establishing a mechanistic link between genome instability and immune activation in lung cancer. Notably, our immune profiling further revealed elevated PD‐L1 expression in Dmap1‐KD tumor cells (Figure S8H), indicating that DNA damage‐induced immune signaling might both stimulate T cell immunity and engage immune checkpoint pathways. These insights extend the understanding of how epigenetic regulation connects DDR with immune modulation, and raise the possibility that exploiting DDR‐immune crosstalk may enhance the existing therapies, such as PARP inhibitors and immune checkpoint blockade. Collectively, our findings highlight an epigenetic‐immune axis that could be leveraged to improve therapeutic strategies against lung cancer.

Nevertheless, several limitations should be noted. First, the activation of intrinsic immunity observed in tumor cells may not fully reflect the multi‐layered interactions that occur in vivo, where stromal and immune cell populations modulate tumor responses. Furthermore, the induction of PD‐L1 suggests that heightened immunogenicity could be offset by checkpoint‐mediated suppression, highlighting the need for combinatorial therapeutic approaches. Third, the underlying mechanism by which Dmap1 regulates replication fork dynamics has not been fully elucidated, and further studies are needed to clarify the causal relationship between Dmap1 knockdown and replication fork dysfunction. As Dmap1 functions as a crucial epigenetic regulator, DNA‐methylation maintainer, and chromatin remodeler [14, 23], we hypothesize that the depletion of Dmap1 impairs proper chromatin assembly ahead of the replication machinery, thereby removing the natural ‘brakes’ on the replisome. This uncoupling between DNA synthesis and histone deposition allows the replication fork to initially travel at an abnormally accelerated speed. However, this unrestrained and uncontrolled over‐speeding is highly unstable. It likely exhausts the local nucleotide pool or exposes excessive ssDNA, inevitably culminating in catastrophic fork stalling and collapse during the subsequent replication phase. Lastly, given the context‐dependent activity of epigenetic regulators, it remains unclear whether DMAP1‐driven immune modulation represents a generalizable phenomenon across cancer types or is restricted to specific oncogenic or genomic contexts. Future studies integrating single‐cell immune profiling, spatial transcriptomics, and co‐culture models will be essential to dissect how DDR‐induced cGAS‐STING signaling intersects with immune response and immune checkpoint regulations. Such mechanistic insights will not only refine our understanding of epigenetic control over tumor‐immune crosstalk but also inform rational strategies for combining DNA damage response‐targeted therapies with immune checkpoint blockade in lung cancer and beyond.

In conclusion, our study uncovers an unexpected oncogenic role of DMAP1 in lung cancer, establishes its previously unrecognized function in maintaining replication‐fork stability, and delineates a mechanistic connection between DMAP1 loss, replication stress‐induced micronuclei, and cGAS‐STING‐driven anti‐tumor immunity. Furthermore, by identifying Dacinostat as a functional DMAP1 suppressor with validated in vivo efficacy, our work highlights a therapeutically actionable strategy targeting DMAP1‐dependent replication and immune pathways.

## 4 Methods

### 4.1 Cell Culture

HEK‐293T cells were cultured in Dulbecco's Modified Eagle Medium (DMEM, MeilunBio, MA0212) with 10% fetal bovine serum (FBS, ExCell Bio, FSP500) and 1% Penicillin‐Streptomycin (PS, NCM Biotech, C100C5). Mouse lung cancer cell lines KP‐1, and human lung cancer cell lines A549 and H2009 were cultured in Roswell Park Memorial Institute (RPMI) 1640 (MeilunBio, MA0215) with 10% FBS and 1% PS (NCM Biotech, C100C5). For in vitro induction of DMAP1/Dmap1 knockdown using pLKO.1‐Tet‐on vector, 100 ng/µL Doxycycline was supplemented in the cell culture medium. All cell lines used in this study were confirmed to be free of mycoplasma contamination by PCR detection.

### 4.2 Plasmid Construction

Plasmids pLKO.1‐Tet‐on‐puro, pCDH‐CMV‐MCS‐EF1‐neo/puro, psPAX2, and pMD2.G were purchased from Addgene. The plasmid pLKO.1‐puro was a kind gift from Fuming Li (Fudan University). All overexpression plasmids of DMAP1 were constructed into pCDH‐CMV‐MCS‐EF1‐neo or pCDH‐CMV‐MCS‐HA‐EF1‐neo/puro using ClonExpress MultiS One Step Cloning Kit (Vazyme, C113). The plasmid pLV3‐ISRE‐minP‐Fluc‐T2A‐EGFP was purchased from MIAOLING PLASMID, and the mmIfnβ mouse minimum promoter and the neomycin eukaryotic resistance were cloned into it using Vazyme C113. All point‐mutations were produced by PCR‐mediated mutagenesis and cloned into the vectors as above. The shRNAs specific for mouse Dmap1 and human DMAP1 were cloned into pLKO.1‐puro and pLKO.1‐Tet‐on‐Puro vector with the AgeI/EcoRI sites. All target sequences are available in Table S1; all constructs were confirmed by Sanger sequencing.

### 4.3 Antibodies and Regents

Western blot antibodies used in this study: DMAP1 (10411‐1‐AP), CDK4 (11026‐1‐AP), Cyclin E2 (85803‐3‐RR), PARP1 (13371‐1‐AP), GAPDH (60004‐1‐Ig), Vinculin (66305‐1‐Ig), β‐Tubulin (66240‐1‐Ig), and β‐Actin (66009‐1‐Ig) antibodies were from Proteintech; Phospho‐Chk2 (Thr68) (HY‐P80799), RPA32/RPA2 (HY‐P80975), Chk1 (HY‐P80082), and Chk2 (HY‐P80083) antibodies were from MCE; Rad51 (F1110), CDK2 (F0022), CDK6 (F0372), Cyclin A2 (F1033), Cyclin B1 (F2522), Cyclin D1 (F2524), Phospho‐CDK1 (Thr161) + CDK2/3 (Thr160) (F3185), Phospho‐CDK1/2/3 (Thr14) (F2120), RPA‐pS33 (F2375), MCM2 (F0581), and PCNA (F0018) antibodies were from Selleck; Phospho‐Chk1 (Ser345) (2348), Phospho‐ATM (Ser1981) (5883), Phospho‐TBK1 (Ser172) (5483), TBK1 (38066), Phospho‐STING (Ser365) (72971), STING (13647), Phospho‐Stat1 (Tyr701) (9167), Stat1 (14994), Phospho‐IRF‐7 (Ser437/438) (24129), IRF‐7 (72073), Phospho‐IRF‐3 (Ser396) (4947), IRF‐3 (4302), HA‐Tag (3724), MAR/PAR (89190), and Phospho‐ATR (Ser428) (2853) antibodies were from CST; CDK1 (A11420) antibody and Rabbit control IgG (AC005) were from Abclonal; Phospho‐CDK6 (Tyr24) (AF2440) and Phospho‐CDK4 (Thr172) (AF8007) antibodies were from Affinity Biosciences; Goat Anti‐Rabbit IgG‐HRP (CW0103) and Goat Anti‐Mouse IgG‐HRP (CW0102) antibodies were from CWBIO.

Reagents and chemicals used in this study inclu CldU (MCE, HY‐112669), IdU (MCE, HY‐B0307), Vadimezan (Selleck, S1537), Nelfinavir (TargetMol, T7779), AG99 (TargetMol, T5155), Nicardipine (TargetMol, T1215), Pantoprazole (TargetMol, T6928), Lidocaine (TargetMol, T1144), Fulvestrant (TargetMol, T2146), Dydrogesterone (TargetMol, T3036), Carbenoxolone (TargetMol, T0992), Metoprolol (TargetMol, T0487), Moxifloxacin (TargetMol, T0331L), Cilomilast (Selleck, S1455), Avasimibe (Selleck, S2187), and Dacinostat (TargetMol, T2454). Elisa kits, including mouse IP‐10/CXCL10 Elisa Kit (E‐EL‐M0021), mouse CXCL16 Elisa Kit (E‐EL‐M0270), and 8‐OHdG Elisa Kit (E‐EL‐0028), were from Elabscience.

### 4.4 Lentivirus Infection

To generate lentivirus, HEK‐293T cells were co‐transfected with lentiviral vectors and packaging plasmids psPAX2 and pMD2.G using PEI (Polysciences, 23966). Viral particles released into the cell culture supernatant, and the supernatant were filtered with 0.45 µm filters (Millipore, SLHVR33RB) to remove cellular debris. Cells were transduced by culturing with viral supernatants in the presence of polybrene (Santa Cruz, sc‐134220) to increase infection efficiency. Stable cell lines were selected and maintained in cell culture media containing 2 µg/mL puromycin, 400 µg/mL G418, or 5 µg/mL Blasticidin S.

### 4.5 Colony Formation Assay

Cells were trypsinized to produce a single‐cell suspension. 500, 1,000, or 2,000 cells were counted and plated in each well of a 6‐well plate. Medium was changed every three days. When the control wells reached adequate confluency, cells were fixed with 70% methanol for 10 min, and cells were stained with 0.5% crystal violet (dissolved in 20% methanol) for 5 min and washed. Photos were taken and quantified using ImageJ.

### 4.6 Cell Apoptosis Assay

The Annexin V‐PE/7‐AAD Apoptosis Detection Kit (Yeasen Biotechnology 40310ES60) was used to measure cell apoptosis by flow cytometry. The cultured cells were harvested at the exponential phase. The data was analyzed by FlowJo v10.8.1.

### 4.7 Cell Cycle Assay

The Cell Cycle and Apoptosis Analysis Kit (Yeasen Biotechnology 40301ES50) was used to measure cell cycle by flow cytometry. The cultured cells were harvested at the exponential phase. The data was analyzed by FlowJo v10.8.1.

### 4.8 Mouse Experiments

Specific‐pathogen‐free facilities were used for housing and care of all mice. Six‐week‐old male C57BL/6 and BALB/c nude mice were purchased from GemPharmatech. CO_2_ inhalation was used to euthanize mice. All animal studies were conducted in compliance with animal protocols approved by the Department of Laboratory Animal Science, Fudan University. All animal experiments were approved by the Experimental Animal Ethics Committee of the School of Basic Medical Sciences, Fudan University (Approval No. 20220228‐079).

For KP‐1 cell subcutaneous models, 1.0 × 10^6^ cells were inoculated into the flanks of C57BL/6 mice. For H2009 cell subcutaneous models, 2.0 × 10^6^ cells were inoculated into the flanks of BALB/c nude mice. Tumor size was measured twice every week using calipers to collect the maximal tumor length and width. Tumor volume was estimated with the following formula: (L × W^2^)/2. Randomization of mouse groups was performed when appropriate.

For lung orthotopic transplantation tumor models, Mice were anesthetized by intraperitoneal injection of xylazine solution at 100 µL per 10 g body weight. After anesthesia, the left chest of the mouse was shaved, and the mouse was placed in a right lateral recumbent position on a surgical pad. The skin on the left chest was disinfected with a cotton ball soaked in 75% ethanol. A 0.5‐1 cm incision was made at the junction of the midline of the left ear base and the lower edge of the axilla. Subcutaneous tissue was bluntly dissected using forceps, and adipose and other tissues were removed to expose the chest wall. The lower edge of the lung was identified and marked as the eighth rib, and, counting upward three ribs to the sixth rib, a needle was inserted at the upper edge of the sixth rib near the midpoint of the incision. A 25 µL cell suspension of 0.25 million KP‐1 cells was drawn into an insulin syringe. The left hand held fine forceps to stabilize the needle at the lower one‐third point, while the right hand inserted the insulin syringe perpendicularly to the point held by the forceps and slowly injected the cells. After injection, the needle was left in place for a few seconds before removal. The injection site was rinsed with PBS and disinfected with a cotton ball soaked in ethanol. The skin was closed using sutures. 3 weeks after tumor inoculation, mice underwent chest CT scanning, and tumor volumes were analyzed using Slicer 5.6.2 software.

### 4.9 Immunohistochemistry (IHC)

For IHC, paraformaldehyde‐fixed tissues were deparaffinized and rehydrated. Antigen was retrieved using 0.01 м sodium‐citrate buffer (pH 6.0) at a sub‐boiling temperature for 15 min, and the sections were incubated with 3% hydrogen peroxide at 37°C for 30 min. After blocking with 5% goat serum at 37°C for 20 min, tissues were incubated with primary antibodies (anti‐Mki67 CST 9129, anti‐CD8a CST 98941S, anti‐CD4 CST 25229S) at 4°C overnight. After three washes in PBS, sections were incubated with an HRP‐conjugated secondary antibody at 37°C for 45 min. Color was developed using DAB Substrate Kit (ZSGB‐BIO, ZLI‐9019), and the counterstaining was carried out with 0.5% hematoxylin. The intensities of Mki67, CD4, and CD8 signals were counted using ImageJ.

### 4.10 Immunofluorescence (IF)

As for immunofluorescence staining on coverslips, the indicated cells were grown on cover slides and fixed with 4% paraformaldehyde for 10 min at room temperature. Then block the specimen in Blocking Buffer (1 × PBS / 5% goat serum / 0.3% Triton X‐100 buffer) for 60 min. Aspirate blocking solution, then incubate with diluted primary antibody (anti‐γ‐H2AX CST 9718S; anti‐β‐actin Proteintech 66009‐1‐Ig) overnight at 4°C. Rinse three times in 1X PBS for 5 min each. Then incubate the specimen in fluorochrome‐conjugated secondary antibody (Goat Anti‐Rabbit IgG (H+L)‐Alexa Fluor 594, Jackson 111‐585‐045; Goat Anti‐Mouse IgG (H+L)‐Alexa Fluor 488, Jackson 115‐545‐062) diluted in Antibody Dilution Buffer for 1–2 h protected from light. After rinsing three times in 1 × PBS for 5 min each, protected from light, samples were mounted with Antifade Mounting Medium with DAPI (Beyotime, P0131) for observation. Images were acquired using a confocal microscope (NOVEL, NCF950).

As for multiplex immunofluorescence assays, paraformaldehyde‐fixed tissues were deparaffinized and rehydrated. Antigen was retrieved using Tris‐EDTA (pH 9.0) at 80°C for 40 min, and the sections were incubated with 3% hydrogen peroxide at 37°C for 10 min. After blocking with 5% BSA at r.t. for 20 min, tissues were incubated with the first primary antibodies (anti‐dsDNA SIGMA ZMS1047, anti‐CD69 Abcam Ab307081, anti‐T‐bet Proteintech 13700‐1‐ap) at 4°C overnight. After three washes in PBS, sections were incubated with fluoresce‐labeled secondary antibody at 37°C for 60 min. After three washes in PBS, tissues were incubated with 100 µL tyramide reagent (try‐594, runnerbio, Bry‐880594) at r.t. for 20 min. After three washes in PBS, the antigen was retrieved using 0.01 м sodium‐citrate buffer (pH 6.0) at a sub‐boiling temperature for 15 min. After three washes in PBS, the same incubation procedure was applied for the second primary antibodies (anti‐CD8a CST 98941, anti‐CD4 CST 25229, anti‐F4/80 CST 70076). After washout of the second antibody, tissues were incubated with 100 µL tyramide reagent (try‐488, runnerbio, Bry‐880488) at r.t. for 20 min. After three washes in PBS, tissues were incubated with 100 µL DAPI (MERCK 10236276001) at r.t. in the dark for 10 min. At last, mount the slides and scan using a digital slide scanner (3DHISTECH, PANNORAMIC SCAN II). For the quantitative analysis, we randomly selected 40 fields at 40 × magnification from similar regions of the tumor on each slide (5 fields per tumor, 8 tumors per slide) and counted the number of positive cells for the respective markers in each field.

### 4.11 Comet Assay

The comet assay was carried out by utilizing the Comet Assay Kit (Beyotime, C2041S) in accordance with the manufacturer's instructions. Pictures were caught and envisaged by a confocal microscope. Tail length and tail DNA percentage were measured using AutoComet software (https://github.com/finkbeiner‐lab/AutoComet); tail moment was calculated as tail moment = (tail length) * (tail DNA percentage).

### 4.12 DNA‐fiber Assay

The DNA fiber assay was carried out following a previously published procedure [49]. Briefly, cells in the exponential growth phase were consecutively incubated with 25 µм CldU and 250 µм IdU (30 min each). Next, cells were harvested, lysed, and spread on microscope slides. After fixation and denaturation, the slides were incubated with primary antibodies (detection of CldU, Abcam ab6326; detection of IdU, Abcam ab181664) for 2 h at room temperature, washed with PBS, and then incubated with secondary antibodies, including Alexa Fluor 594 (Jackson 112‐585‐003) and Alexa Fluor 488 (Jackson 115‐545‐062), and analyzed using the ImageJ software.

### 4.13 iPOND Assay

iPOND was performed as previously described [50]. Briefly, logarithmically growing KP‐1 cells (∼ 10^8^ cells) were labelled with 10 µм EdU (TargetMol, T17341) for 10 min. Cells were then fixed with 1% formaldehyde for 20 min, quenched with 1.25 м glycine, and collected in 50 mL tubes. Cells were then permeabilized with 0.25% triton X‐100 at r.t. for 30 min. Cells were split, one for the ‘click’ reactions to conjugate biotin to the EdU‐labelled DNA, and the other as the negative control by replacing biotin azide (TargetMol, T41046) with DMSO for a mock click reaction. Briefly, the cell pellets were resuspended with click reaction mix (a 5 mL click reaction mix was for ∼ 10^8^ cells) and incubated at r.t. for 1 h. The samples were collected and 600 µL lysis buffer with protease inhibitors was added to each sample for sonication, followed by centrifugation at the maximum speed for 10 min at r.t. The supernatants were diluted with equal volume of PBS, 50 µL of which was used as input. Streptavidin beads (Beyotime, P2151) were then used to capture the biotin‐conjugated DNA‐protein complexes overnight at 4°C. Purified replication fork proteins were eluted by boiling in SDS‐sample buffer, and one‐tenth of which was subjected to western blotting analysis.

### 4.14 Co‐IP and IP‐MS

KP‐1 cells were lysed with Cell lysis buffer for Western and IP (Beyotime, P0013), and then protein concentration was determined by BCA assay (Beyotime, P0011). The cell extracts were used for immunoprecipitation with Dmap1 antibody or Rabbit control IgG. In detail, 3 µg antibody was added to the 1500 ug supernatant and incubated at 4°C overnight. Then, 25 µL Protein A+G Magnetic Beads (Beyotime, P2108) were added, and the sample was incubated for a further 4 h at 4°C. The beads were washed in TBST buffer (50 mм Tris‐HCl, pH 7.4, 150 mм NaCl, 0.1% TWEEN 20) five times and eluted with SDS buffer (50 mM Tris, pH 6.8, 150 mmol/L NaCl, 2% SDS, 10% glycerin; 5% β‐mercaptoethanol, 0.05% bromophenol blue). 20 µg protein for each sample was mixed with 2X loading buffer, respectively, and boiled for 5 min. The proteins were separated on an SDS‐PAGE gel (constant current 120 V, 60 min). For co‐IP assays, a western blot was performed. For IP‐MS experiments, protein bands were visualized by Coomassie Blue R‐250 staining. Target Protein bands were collected, and digestion was performed by trypsin.

After the gel pieces underwent destaining and fragmentation, a solution containing 20 ng/µL trypsin in 50 mM NH4HCO3 was added. Enzymatic digestion took place in a 37°C incubator for 16 h, followed by the extraction and freeze‐drying of peptide extracts. Prior to analysis, the samples were resolubilized in a 0.1% formic acid solution and underwent peptide quantification before being subjected to mass spectrometry analysis.

For the proteome profiling samples, peptides were analyzed on a Q Exactive HF‐X Hybrid Quadrupole‐Orbitrap Mass Spectrometer (Thermo Fisher Scientific) coupled with a high‐performance liquid chromatography system (EASY nLC 1200, Thermo Fisher Scientific). Dried peptide samples re‐dissolved in Solvent A (0.1% formic acid in water) were loaded onto a 2‐cm self‐packed trap column (100 µm inner diameter, 3 µm ReproSil‐Pur C18‐AQ beads, Dr. Maisch GmbH) using Solvent A and separated on a 150‐µm‐inner‐diameter column with a length of 15 cm (1.9 µm ReproSil‐Pur C18‐AQ beads, Dr. Maisch GmbH) over a 30‐min gradient (Solvent A: 0.1% formic acid in water; Solvent B: 0.1% formic acid in 80% ACN) at a constant flow rate of 600 nL/min (0–30 min, 0 min, 8% B; 0–4 min, 8–15% B; 4–19 min, 15–30% B; 19–22 min, 30–50% B; 22–23 min, 50–100% B; 23–30 min, 100% B). Eluted peptides were ionized at 2 kV and introduced into the mass spectrometer. Mass spectrometry was performed in data‐dependent acquisition mode. For the MS1 Spectra full scan, ions with m/z ranging from 300 to 1400 were acquired by an Orbitrap mass analyzer at a high resolution of 120,000. The automatic gain control (AGC) target value was set to 3E+06. The maximal ion injection time was 80 ms. The top 60 precursor ions were selected for fragmentation in an HCD cell with a normalized collision energy of 27%. The resulting fragment ions were transferred to the Orbitrap analyzer, which operated at a resolution of 7500. The automatic gain control (AGC) was set to 5e4 for MS/MS. The maximum ion injection times were set to 20 ms. Dynamic exclusion of previously acquired precursor ions was enabled for 15 s. The original data of mass spectrometry analysis were RAW files, and Proteome Discoverer 2.3 was used for qualitative and quantitative analysis.

For IP‐MS data analysis, to identify Dmap1 interactors, a protein was defined as an interacting partner if it met at least one of the following three criteria: (1) Detected qualitatively in the IP group (lost quantification) but not detected in the IgG control group (lost detection); (2) Detected quantitatively in the IP group, but not detected or detected qualitatively (lost quantification) in the IgG group; (3) Quantitatively detected in both groups, and the protein exhibited more than a two‐fold higher abundance in the IP group compared to the IgG control. Dmap1 interacting partners were then clustered using the R package STRINGdb 2.20.0, and PPI plots were visualized using Cytoscape 3.10.0.

### 4.15 Tumor‐infiltrating Immune Cell Isolation and FACS Analysis

Mice were euthanized. Subcutaneous tumors were minced and digested in collagenase D (Nordmark, S1745401) and DNase I (Sigma–Aldrich, 10104159001) in Hank's Balanced Salt Solution at 37°C for 30 min. After incubation, digested tissue was filtered through a 70‐µm cell strainer to obtain single‐cell suspensions. Separated cells were treated with RBC lysis buffer (TIANGEN, RT122‐02) to lyse red blood cells. Live cells were determined with a Zombie Aqua Fixable Viability Kit (BioLegend, 423102). Cell pellets were resuspended in PBS with 2% FBS for FACS analysis. Cells were stained with the indicated cell‐surface markers and fixed/permeabilized using an eBioscience Foxp3/Transcription Factor Staining Buffer Set (Invitrogen, 00‐5523‐00). After that, cells were stained with the indicated intracellular markers and resuspended. Cells were acquired on a BD FACSAria III and analyzed with FlowJo v10.8.1. The gating strategy was described in Figure S6A.

### 4.16 Flow Antibodies

Subcutaneous tumor‐infiltrating tumor and immune cells were stained with fluorochrome‐conjugated antibodies against mouse CD45 (BioLegend, 103139), CD4 (BioLegend, 100552), CD8 (BioLegend, 100742), CD44 (BioLegend, 103032), CD62L (BioLegend, 104412), CD69 (BioLegend, 104536), ICOS (BioLegend, 313530), CD11B (BioLegend, 101259), CD11C (BioLegend, 117336), Ly6G (BioLegend, 127618), GR1 (BioLegend, 108452), CD103 (BioLegend, 121414), F4/80 (BioLegend, 123128), CD80 (BioLegend, 104712), CD86 (BioLegend, 105030), IA/IE (BioLegend, 107626), IFNγ (BioLegend, 505826), GZMB (BioLegend, 372216), CD206 (BioLegend, 141717), EpCam (BioLegend, 118208), H2 (BioLegend, 125506), and PD‐L1 (BioLegend, 124324). For H2 and H2‐bound Ova analysis, indicated cells were stimulated with mouse IFNγ, and then stained with fluorochrome‐conjugated antibodies against H2 (BioLegend, 125506), H2 bound to SIINFEKL (BioLegend, 141606), and corresponding isotypes (BioLegend, 400507 and 400120).

### 4.17 Western Blot

Cells were lysed with SDS buffer (62.5 mм Tris, pH 6.8, 150 mм NaCl, 2% SDS, 10% glycerin; 5% β‐mercaptoethanol, 0.05% bromophenol blue) and protein concentration was determined by BCA assay (Beyotime, P0011). Samples were boiled for 10 min, and 10 to 40 µg of protein was separated by SDS‐PAGE, transferred to polyvinylidene difluoride (PVDF) membranes (Millipore, ISEQ00010) according to standard protocols, and probed with the relevant primary antibodies overnight at 4°C. Membranes were then incubated with HRP–conjugated anti‐rabbit IgG or anti‐mouse IgG secondary antibodies at room temperature for 2 h, and proteins were detected using Pierce ECL Western Blotting Substrate (Abclonal, RM00021). ECL‐developed blots were imaged using a Touch Imager (e‐BLOT).

### 4.18 RNA‐seq and Data Analysis

Cell total RNA was harvested using TRNzol Universal Reagent (TIANGEN #DP424). RNA purity and concentration were then examined using NanoDrop 2000; RNA integrity and quantity were measured using the Agilent 2100/2400 system. RNA libraries were prepared at Berry Genomics as follows: mRNA was purified from total RNA using polyT and then fragmented into 300–350 bp fragments, the first strand cDNA was reverse‐transcribed using fragmented RNA and dNTPs (dATP, dTTP, dCTP, and dGTP) and second strand cDNA synthesis was subsequently performed. Remaining overhangs of double‐strand cDNA were converted into blunt ends via exonuclease/polymerase activities. After adenylation of 3' ends of DNA fragments, sequencing adaptors were ligated to the cDNA and the library fragments were purified. The template was enriched by PCR, and the PCR product was purified to obtain the final library.

For data analysis, the raw read counts quality was checked using fastqc v0.12.1. Then, sequence reads were pseudo‐aligned to GrCM38/mm10 using Kallisto v0.50.1 (parameters—default parameter, ‐t 10). Read count normalization and downstream analysis were performed using edgeR v4.6.3 and limma v3.64.1 in R v4.5.1. Gene set enrichment analysis was done using GSEA (v.3.0) and gene sets from MSigDB (v.5.0). We used the ‘preranked’ algorithm to analyze gene lists ranked by the negative decadic logarithm of *p* values multiplied by the value of log2FC obtained from the differential‐expression analysis with limma.

### 4.19 CUT&Tag and Data Analysis

CUT&Tag experiment was conducted using the CUT&Tag kit complemented with Hyperactive TF Enhancer Module from Vazyme (TD904‐C2). Briefly, cells were collected and treated with the TF enhancer. Subsequently, cells were bound with Concanavalin A‐coated magnetic beads, and the plasma membrane was permeabilized with digitonin. Through the use of a primary antibody targeting HA (CST, 3724) or a non‐targeting IgG control (Abclonal, AC042), a corresponding secondary antibody, and Protein A/G, the transposase fused to Protein A/G was guided to precisely cleave DNA sequences near the target protein. During cleavage, adaptor sequences were added to both ends of the DNA fragments. As for CUT&Tag‐qPCR, the DNA products were released at this step using the CUT&Tag Stop Buffer for qPCR (TD904‐C1) and subsequently analyzed by RT‐qPCR using promoter region primers (Table S3); as for CUT&Tag‐seq, CUT&Tag libraries were prepared using TD904‐C2 and TruePrep Index Kit V2 for Illumina (TD202) from Vazyme. All of the cleaved DNA extracted using the CUT&Tag kit was used as starting material for anti‐HA and anti‐IgG samples. Before PCR amplification, 0.05 pg DNA Spike‐in was added into each sample as a reference standard, primarily for inter‐sample normalization of the sequencing data. Libraries were then amplified using 16 cycles on the thermocycler. Libraries were validated using the Fragment Analyzer 5300 system.

For CUT&Tag‐qPCR data analysis, we used the 2^−ΔΔCT^ method. The detailed workflow was as follows: (1) Quantify the CT values of the target gene and the DNA spike‐in in the treatment group, control group, and IgG negative control; (2) Using the DNA spike‐in as the internal reference, calculate the ΔCT values for each group: Treatment group: ΔCT_treatment = CT_treatment − CT_treatment (DNA spike‐in); Control group: ΔCT_control = CT_control − CT_control (DNA spike‐in); Negative IgG control: ΔCT_IgG = CT_IgG − CT_IgG (DNA spike‐in); (3) Using the IgG negative control as the reference, calculate the 2^‐ΔΔCT values for each group: Relative Enrichment_fold_treatment = 2^−(ΔCT_treaent − ΔCT_IgG)^; Relative Enrichment_fold_control = 2^−(ΔCT_control − ΔCT_IgG)^; Relative Enrichment_fold_IgG = 2^−(ΔCT_IgG − ΔCT_IgG)^ = 1.

For CUT&Tag‐seq data analysis, low quality reads and sequencing adaptors of the CUT&Tag reads were trimmed using fastp v0.24.0 with the parameter of “‐T 1 ‐5 ‐l 50 ‐q 20 ‐u 50 ‐g ‐c –detect_adapter_for_pe ‐w 9”. CUT&Tag reads were then local‐aligned to the mouse mm10 genome and the DNA Spike‐in sequence separately using bowtie2 v2.5.4 with the parameter of “‐p 9 ‐q –local –very‐sensitive –no‐mixed –no‐discordant –phred33 ‐I 10 ‐X 700”. Scaling factors of each sample were calculated as 10,000 / (fragments mapped to Spike‐in sequence). Normalized coverage for each sample was calculated as the primary genome coverage multiplied by the scaling factor. Peaks were called using MACS v3.0.3 with the significance cut‐off q‐value ≤ 0.01. BigWig files were generated using the bamCoverage function of deeptools v3.5.6. Score of the BigWig files represents the normalized coverage of DNA fragments at a given genomic coordinate.

### 4.20 Single‐cell RNA‐seq and Data Analysis

We inoculated wild‐type (shLacZ) and Dmap1 knockdown (shDmap1) KP‐1 cells into C57Bl/6 mice subcutaneously. Following the acquisition of samples from the subcutaneous tumor, we carried out single‐cell sequencing (scRNA‐seq) on the tumors at Singleron as follows: single‐cell suspensions (2 × 10^5^ cells/mL) with PBS (HyClone) were loaded onto a microwell chip using the Singleron Matrix Single Cell Processing System. Barcoding Beads were subsequently collected from the microwell chip, followed by reverse transcription of the mRNA captured by the Barcoding Beads and to obtain cDNA, and PCR amplification. The amplified cDNA was then fragmented and ligated with sequencing adapters. After that, the scRNA‐seq libraries were constructed according to the protocol of the GEXSCOPE Single Cell RNA Library Kits (Singleron). Individual libraries were diluted to 4 nм, pooled, and sequenced on Illumina Novaseq 6000 with 150 bp paired‐end reads.

For data analysis, raw reads from scRNA‐seq were processed to generate gene expression matrices using the CeleScope (https://github.com/singleron‐RD/CeleScope) v1.9.0 pipeline. Briefly, raw reads were first processed with CeleScope to remove low‐quality reads with Cutadapt v1.17 to trim poly‐A tail and adapter sequences. Cell barcode and UMI were extracted. After that, we used STAR v2.6.1a to map reads to the reference genome GRCm38/mm10. UMI counts and gene counts of each cell were acquired with featureCounts v2.0.1 software and used to generate expression matrix files for subsequent analysis. Downstream analysis was performed by Seurat v4.4.0 in R v4.5.1. SCP v0.5.6 and scRNAtoolVis v0.1.0 were used for data visualization.

### 4.21 Quantitative RT‐PCR

Total RNA was extracted from cells using the TRNzol Universal kit (TIANGEN, DP424), and cDNA was generated with a HiScript III RT SuperMix for qRT‐PCR (+gDNA wiper) Kit (Vazyme, R323). Quantitative RT‐PCR was performed using SYBR Green PCR Master Mix (Vazyme, Q341), and transcript levels were normalized to Actin as an internal control. Samples were run in triplicate. Primers for the corresponding genes are available in Table S2.

### 4.22 Inhibitor Prediction

The L1000 platform was used for inhibitor prediction (clue.io). The top 150 up‐regulated and top 150 down‐regulated genes were obtained from KP‐1 RNA‐seq data, and were converted to human orthologs as input. Candidates were ranked by the “norm_score” column of the results. The top 13 drugs that had undergone clinical trial or received FDA approval were selected for experimental validation.

### 4.23 Statistical Analysis

All statistical analyses were carried out using GraphPad Prism 8 or R v4.5.1. Estimate v1.0.13 was used for ESTIMATE analysis. TCGAplot v8.0.0 was used for data visualization of the TCGA datasets. Data was analyzed by Student's t‐test (two tailed) unless otherwise specified. *P* < 0.05 was considered statistically significant. Error bars represent the standard error of the mean (SEM).

## Author Contributions

F. L., Y. S., K. H., X. D., S. L., and C. L. conceived the project and research plan. K. H., X. D., S. L., Y. C., Y. Y., and C. L. performed functional and mechanistic studies. K. H. performed bioinformatic data analysis, including RNA‐seq, CUT&Tag, and single‐cell RNA‐seq analysis. L. W. performed the lung in situ tumor inoculation experiment. K. L. provided technical support and many constructive suggestions on bioinformatic analysis. S. L., Y. C., and C. L. provided useful suggestions on figure packaging and optimization. K. H., F. L., Y. S., and C. L. wrote the manuscript. S. L. provided useful suggestions on manuscript revision.

## Funding

This work was supported by the National Basic Research Program of China (2022YFA1103900), National Natural Science Foundation of China (82372794, 82572985, 82172744, 82503107), Shanghai medical key specialty construction plan (ZK2019C14), the Postdoctoral Fellowship Program of CPSF (GZB20240544), the China Postdoctoral Science Foundation (2024M752432), the Natural Science Foundation of Shanghai (24ZR1469100).

## Conflicts of Interest

The authors declare no conflicts of interest.

## Supporting information




**Supporting File**: advs75020‐sup‐0001‐SuppMat.docx.

## Data Availability

High‐throughput sequencing datasets generated in this study have been deposited in the National Center for Biotechnology Information's Gene Expression Omnibus: RNA‐seq datasets are accessible through GEO Series accession number GSE304428 (https://www.ncbi.nlm.nih.gov/geo/query/acc.cgi?acc = GSE304428); CUT&Tag datasets are accessible through GSE304430 (https://www.ncbi.nlm.nih.gov/geo/query/acc.cgi?acc = GSE304430); Single‐cell RNA‐seq datasets are accessible through GSE304429 (https://www.ncbi.nlm.nih.gov/geo/query/acc.cgi?acc = GSE304429). The CRISPR screen datasets were accessible through GSE127232 as previously described [22]. NSCLC cohort data used for estimate and survival analysis were sourced from GSE37745. Other data used in this study were sourced from The Cancer Genome Atlas data on UCSC Xena (http://xena.ucsc.edu), and The Human Protein Atlas (https://www.proteinatlas.org), both of which are publicly accessible datasets.
