# Supplementary material for: DMAP1 Deficiency Suppresses Lung Cancer Progression by Destabilizing Replication Fork and Activating IFN Signaling‐Mediated Anti‐tumor Immunity
Source: Adv Sci (Weinh). 2026 Mar 29;13(33):e17634. doi: 10.1002/advs.202517634 (PMC13271618; doi:10.1002/advs.202517634)
Supplement: Supplementary file 1 — Supporting File: advs75020‐sup‐0001‐SuppMat.docx. [file ADVS-13-e17634-s001.docx]

Supporting Information

**DMAP1 Deficiency Suppresses Lung Cancer Progression by Destabilizing Replication Fork and Activating IFN Signaling-Mediated Anti-Tumor Immunity**

*Kan Huang, Xi Dai, Shuaihu Li, Yingxue Chen, Yaxin Yu, Lin Wang, Kun Liu, Shuhan Lyu, Chongyang Li,^*^ Yihua Sun,^*^ and Fei Li ^*^*

**
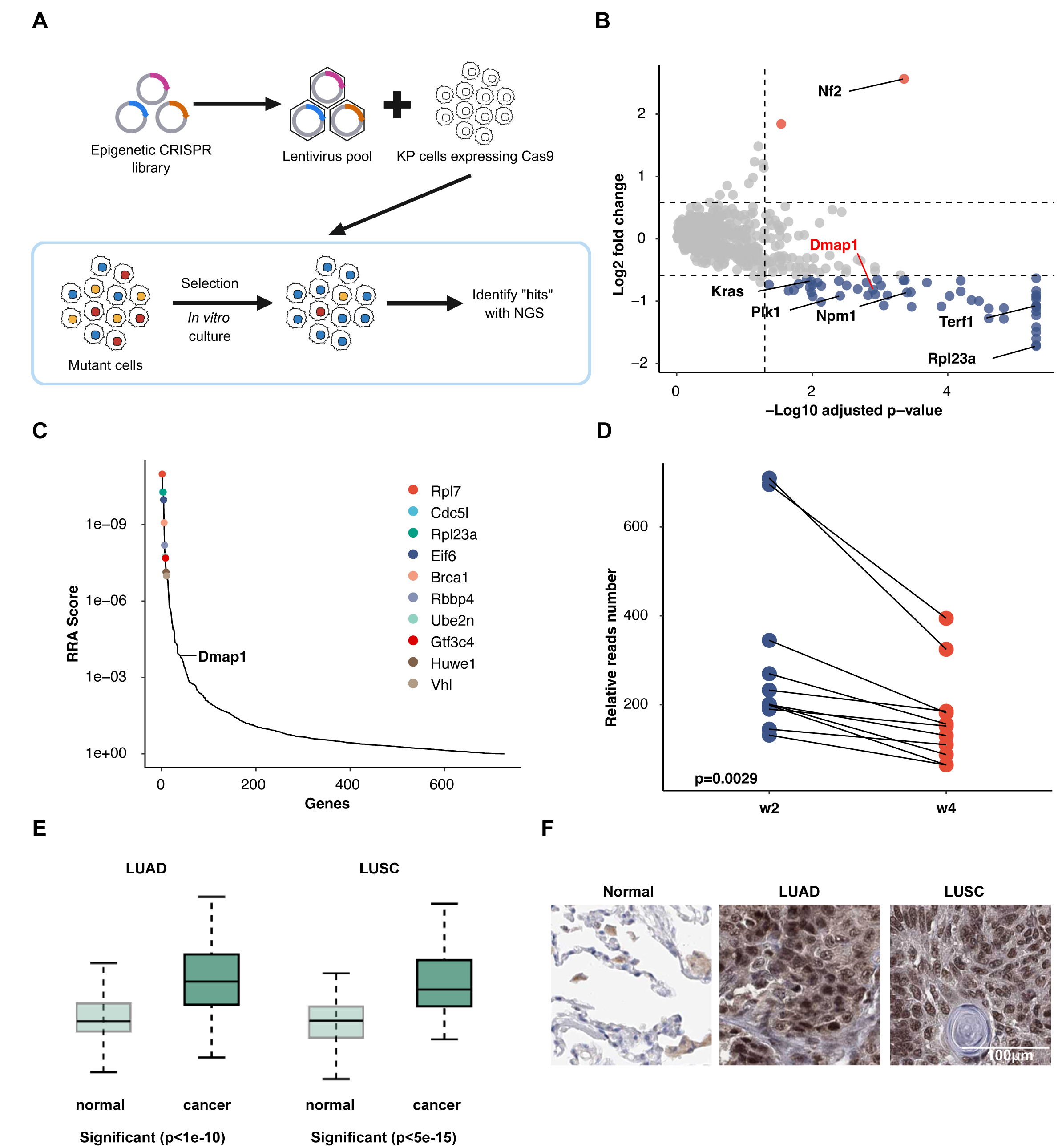
**

**Figure S1.** **DMAP1 is crucial in the progression of lung cancer.** **A**, Strategy for epigenetic-focused CRISPR screening. Created in BioRender. Huang, K. (2026) https://BioRender.com/59asvaz. **B**, Volcano plot of comparison between the week 2 and the week 4 clone 9 KP-1 cells from the screen. **C**, RRA plot for the top candidates and Dmap1 of the screen in clone 9 KP-1 cells. **D**, Performance of sgRNAs targeting Dmap1 in clone 9 KP-1 cells of the screen, n = 11. **E**, The comparison of DMAP1 expression in The Human Protein Atlas (HPA) cohorts of lung cancers. **F**, Representative fields of IHC staining of DMAP1 in normal lung, lung adenocarcinoma and lung squamous cell carcinoma from the HPA database. Data were analyzed using two-tailed paired t-test [(B)], and two-tailed t-test [(E)]. Figure S1 A-D were reproduced with permission.^[1]^


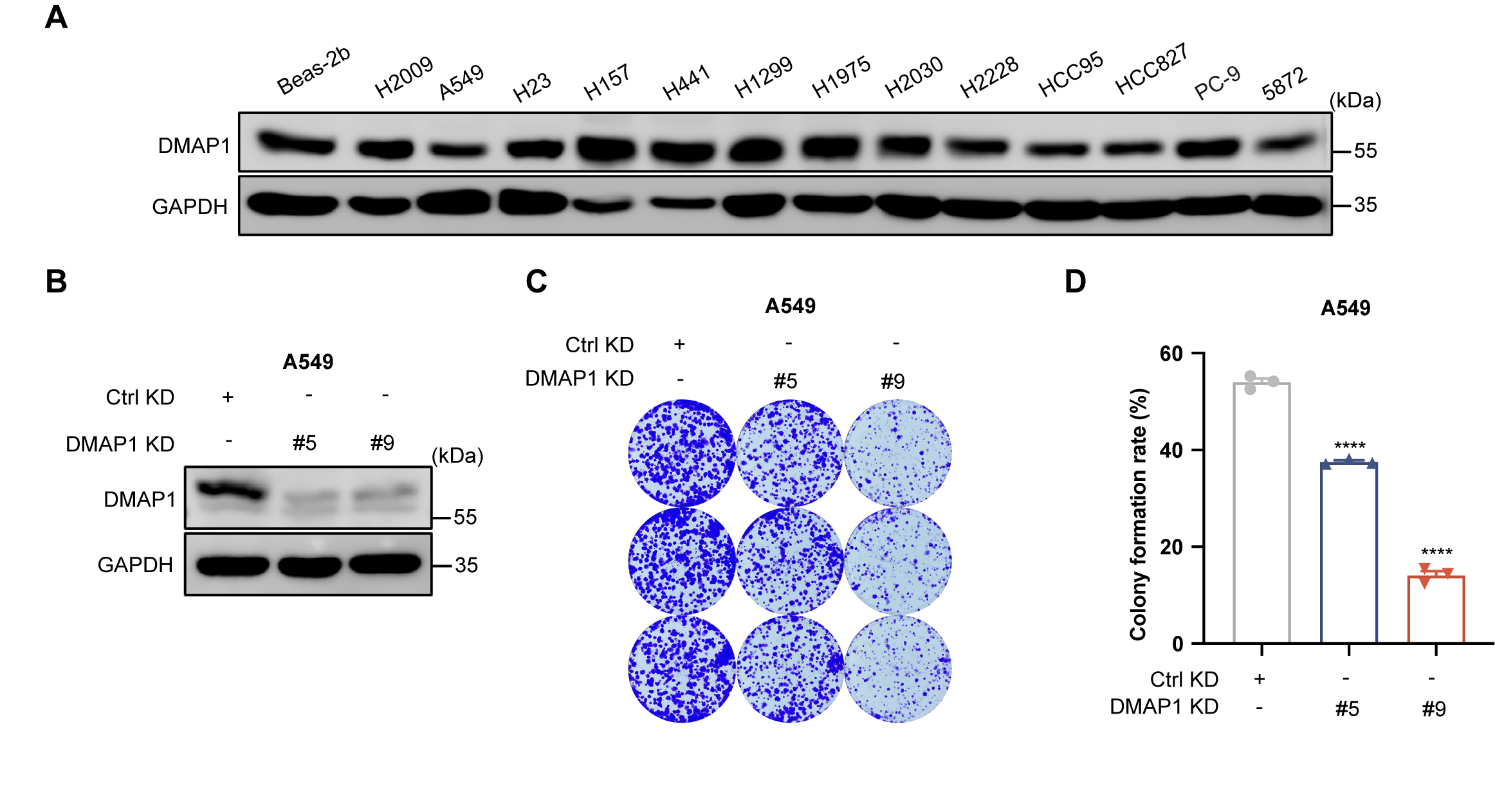


**Figure S2. Dmap1 knockdown specifically attenuates tumor cell growth. A**, DMAP1 expression level in Beas-2b and 13 NSCLC cell lines. **B**, Western blot of DMAP1 knockdown in A549 cells. **C**, Colony formation assays of A549-shGFP or shDMAP1 cells. **D**, Statistical analysis for (**C**), n = 3. Data were analyzed using two-tailed t-test. ****, P < 0.0001.


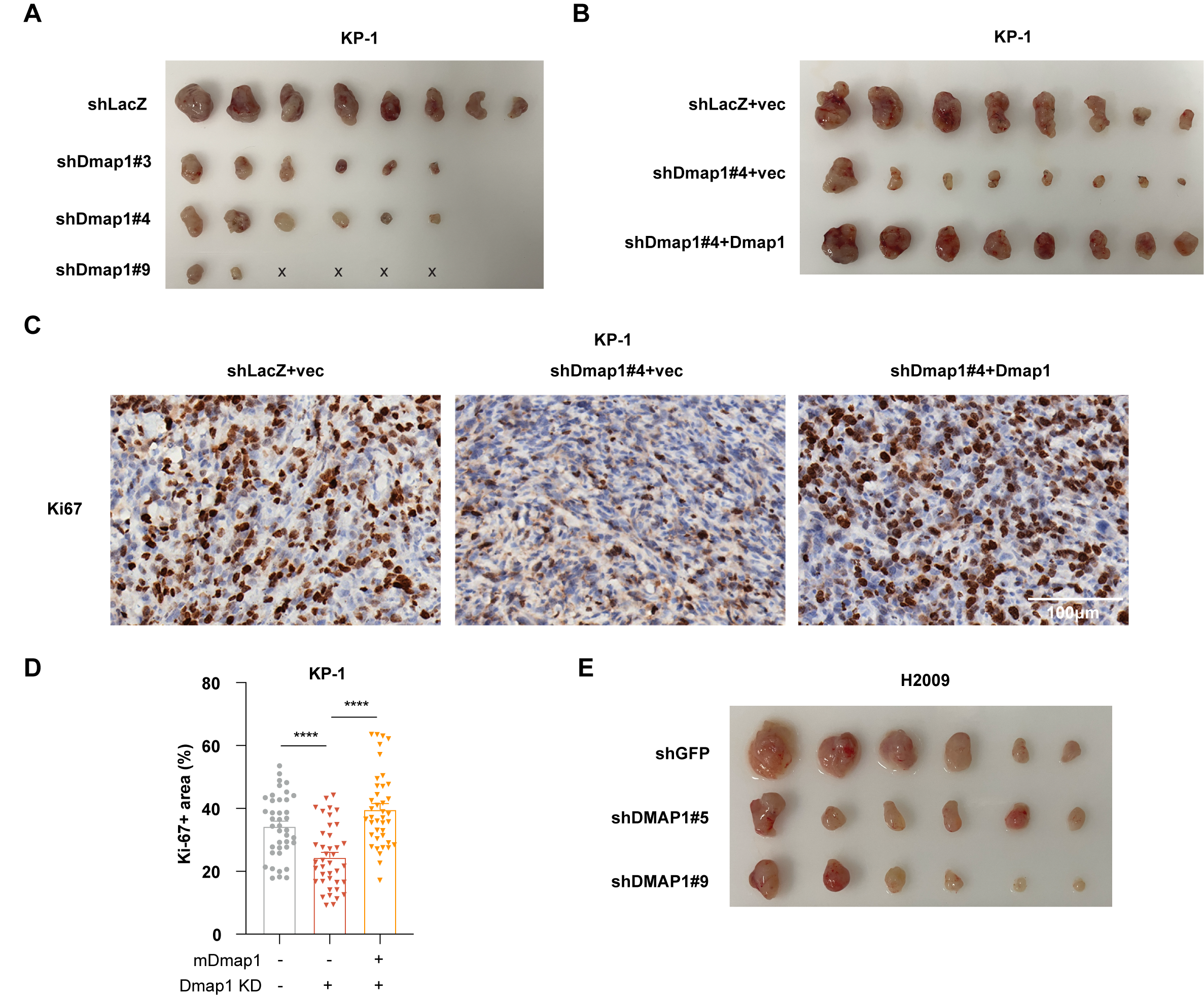


**Figure S3. Dmap1 knockdown reduces tumor cell growth *in vivo*. A-B**, Tumor photo for Figure 2A (**A**) and Figure 2C (**B**). **C**, Representative fields of IHC staining in KP-1 C57BL/6 subcutaneous tumor samples stained for Ki67. **D**, Statistical analysis for (**C**), n = 40. **E**, Tumor photo for Figure 2I. Data are presented as mean ± SEM. Data were analyzed using two-tailed t-test. ****, P < 0.0001.


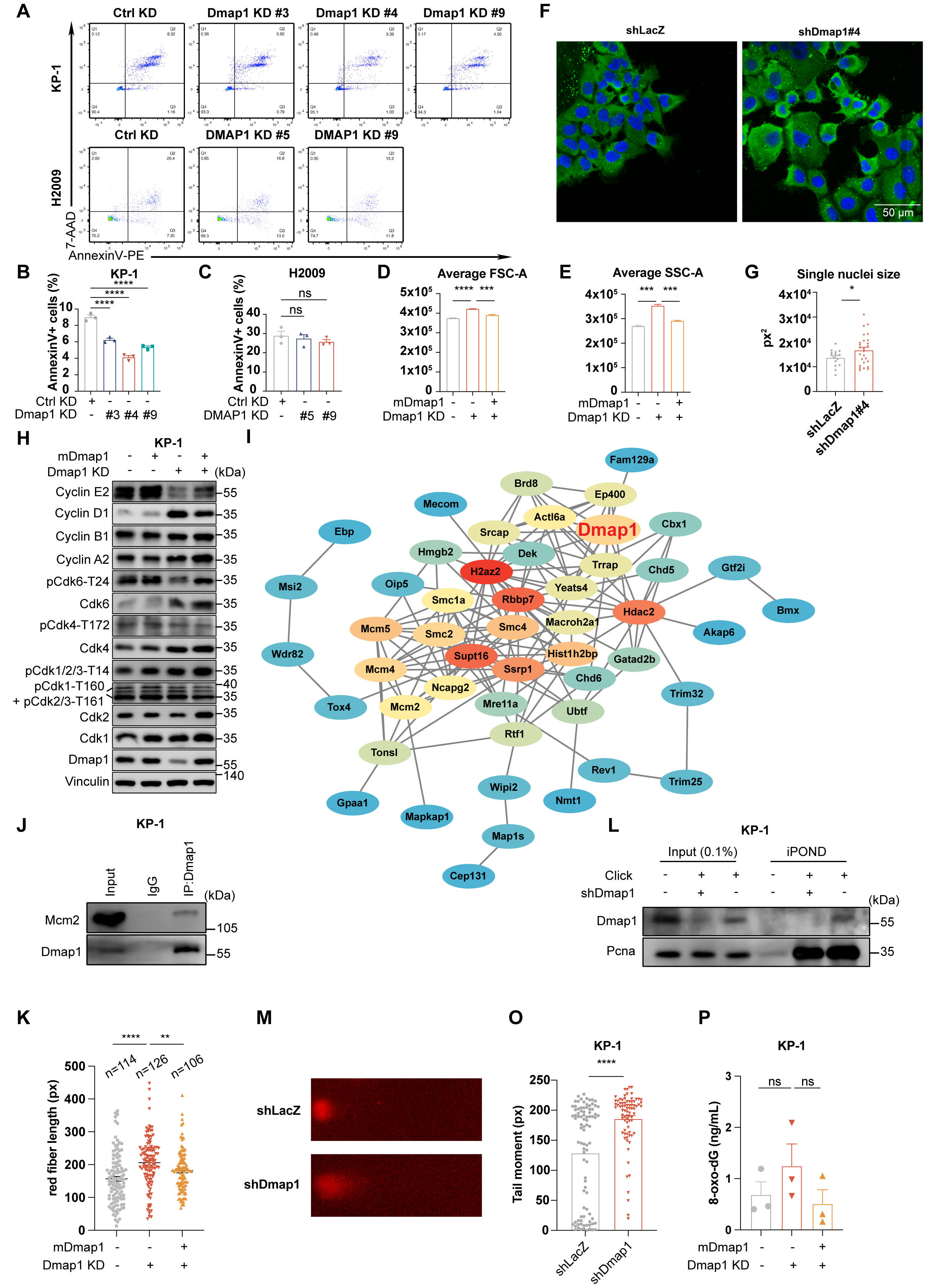


**Figure S4. Dmap1 knockdown does not promote tumor cell apoptosis but caused cell cycle arrest and DNA damage. A**, Representative flow cytometry analysis of cell apoptosis in KP-1 and H2009 cells. **B-C**, Statistics for (**A**), t-test, n = 3. **D-E**, Quantitative analysis of average FSC-A (**D**) and SSC-A (**E**) of KP-1 cells in the flow cytometry analysis in Figure 3D-E, t-test, n = 3. **F**, Representative field of KP-1 shLacZ and shDmap1#4 cells stained with β-actin (green) and DAPI (blue). **G**, Quantitative analysis by ImageJ of the size of the single nuclei in (**F**), Welch’s t-test, n = 15 (shLacZ), 26 (shDmap1#4). **H**, KP-1 cells with Dmap1 WT, OE, KD, and rescue cells were analyzed using CDK and cyclin-related antibodies. **I,** PPI plot for Dmap1-interacting proteins. **J**, Co-IP assay of the interaction between Dmap1 and Mcm2. **K**, Statistical analysis of the absolute CldU (red) fiber length for Figure 3G, t-test. **L**, iPOND assay of the replication-fork interacting protein, Pcna was used as a positive control, and biotin-azide free samples (no click) was used as negative controls. **M**, Representative “comet” for comet assay in KP-1 cells. **O**, Statistics for (**M**), Mann-Whitney test, shLacZ, n = 90, shDmap1, n = 79. **P**, Statistics for 8-oxo-dG Elisa assay, t-test, n = 3. ns, not significant; *, P < 0.05; ****, P < 0.0001.


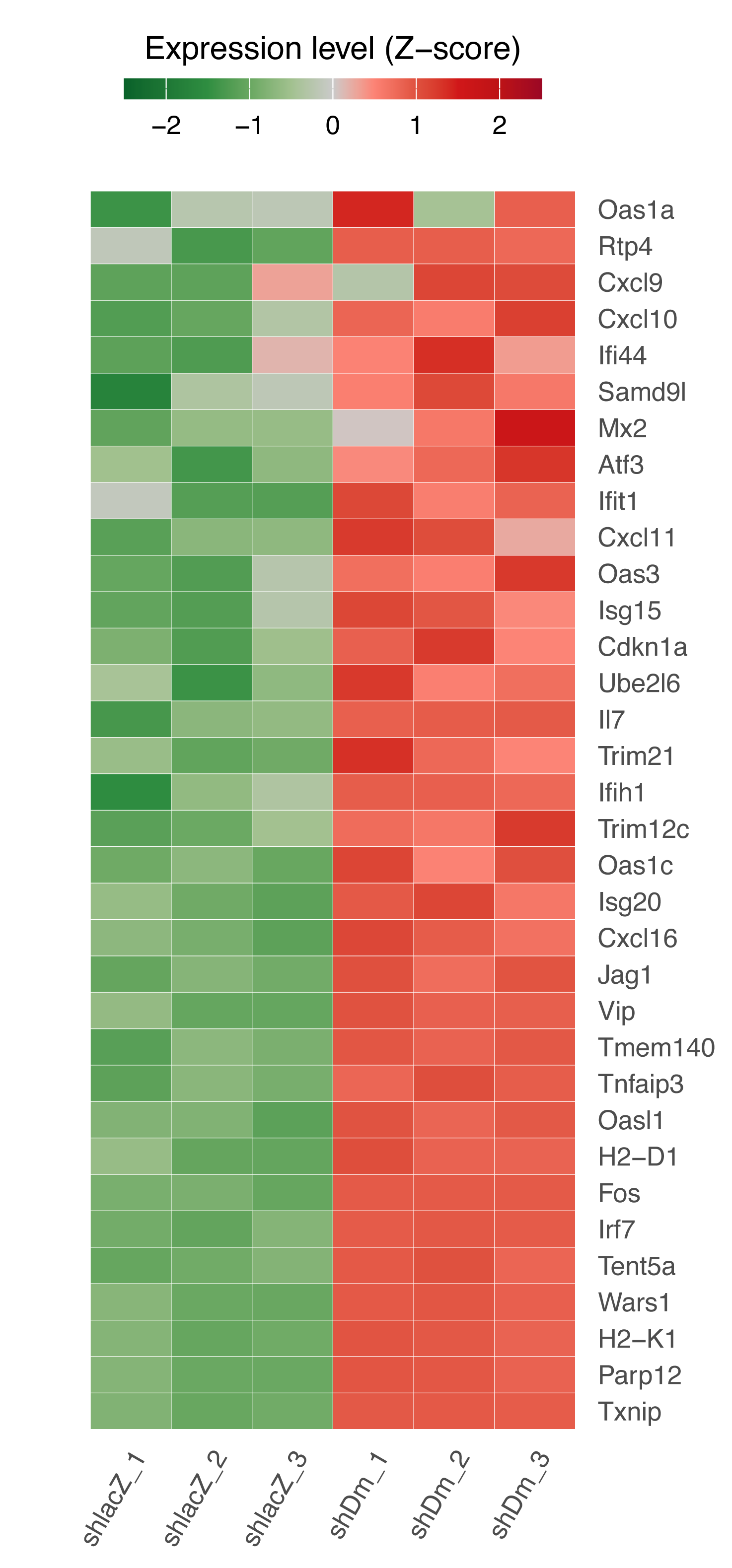


**Figure S5. Heatmap shows the ISGs upregulated in shDmap1 group in the RNA-seq data.**

**
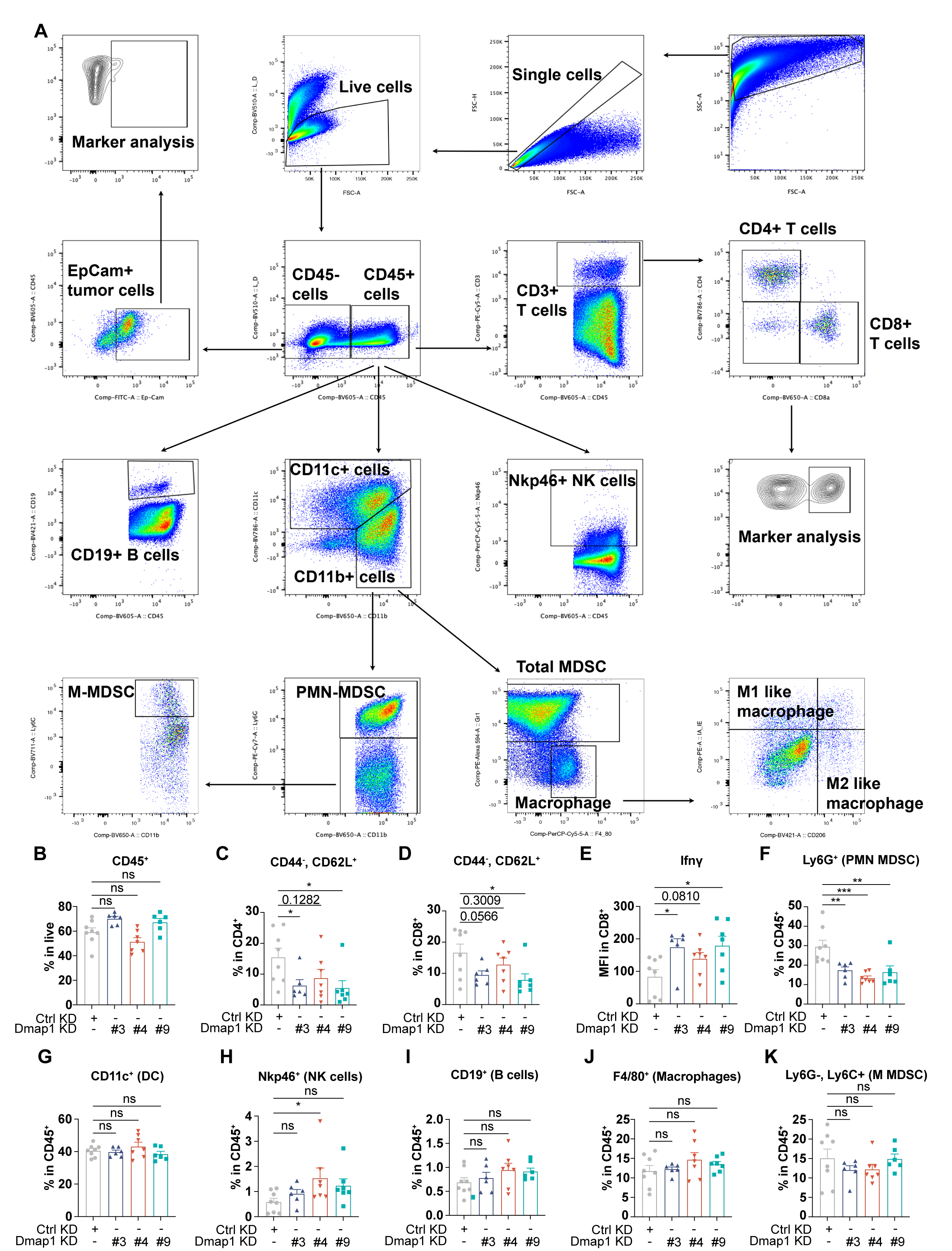
**

**Figure S6. Gating strategy of the flow analysis and immune cell groups abundance statistics. A,** Gating strategy for immune profile analysis in this study. **B**, Flow analysis of changes of CD45^+^ populations, n = 8 (Ctrl KD), 6 (shDmap1#3), 7 (shDmap1#4), and 7 (shDmap1#9). **C-D,** Flow analysis of changes of CD44^-^, CD62L^+^ populations in CD4^+^ (**C**) and CD8^+^ (**D**) cells; n = 8 (Ctrl KD), 6 (shDmap1#3), 7 (shDmap1#4), and 7 (shDmap1#9). **E**, Flow analysis of changes of Ifnγ MFI in CD8^+^ cells; n = 8 (Ctrl KD), 6 (shDmap1#3), 7 (shDmap1#4), and 7 (shDmap1#9). **F-K**, Flow analysis of Ly6G^+^ (**F**), Cd11c^+^ (**G**), Nkp46^+^ (**H**), CD19^+^ (**I**), F4/80^+^ (**J**), and M-MDSC (**K**) populations in CD45^+^ immune cells; F, G, K, n = 8 (Ctrl KD), 6 (shDmap1#3), 7 (shDmap1#4), and 6 (shDmap1#9); H-J, n = 8 (Ctrl KD), 6 (shDmap1#3), 7 (shDmap1#4), and 7 (shDmap1#9). Data are presented as mean ± SEM. Data were analyzed using two-tailed t-test. ns, not significant; *, P < 0.05; **, P < 0.01; ***, P < 0.001.


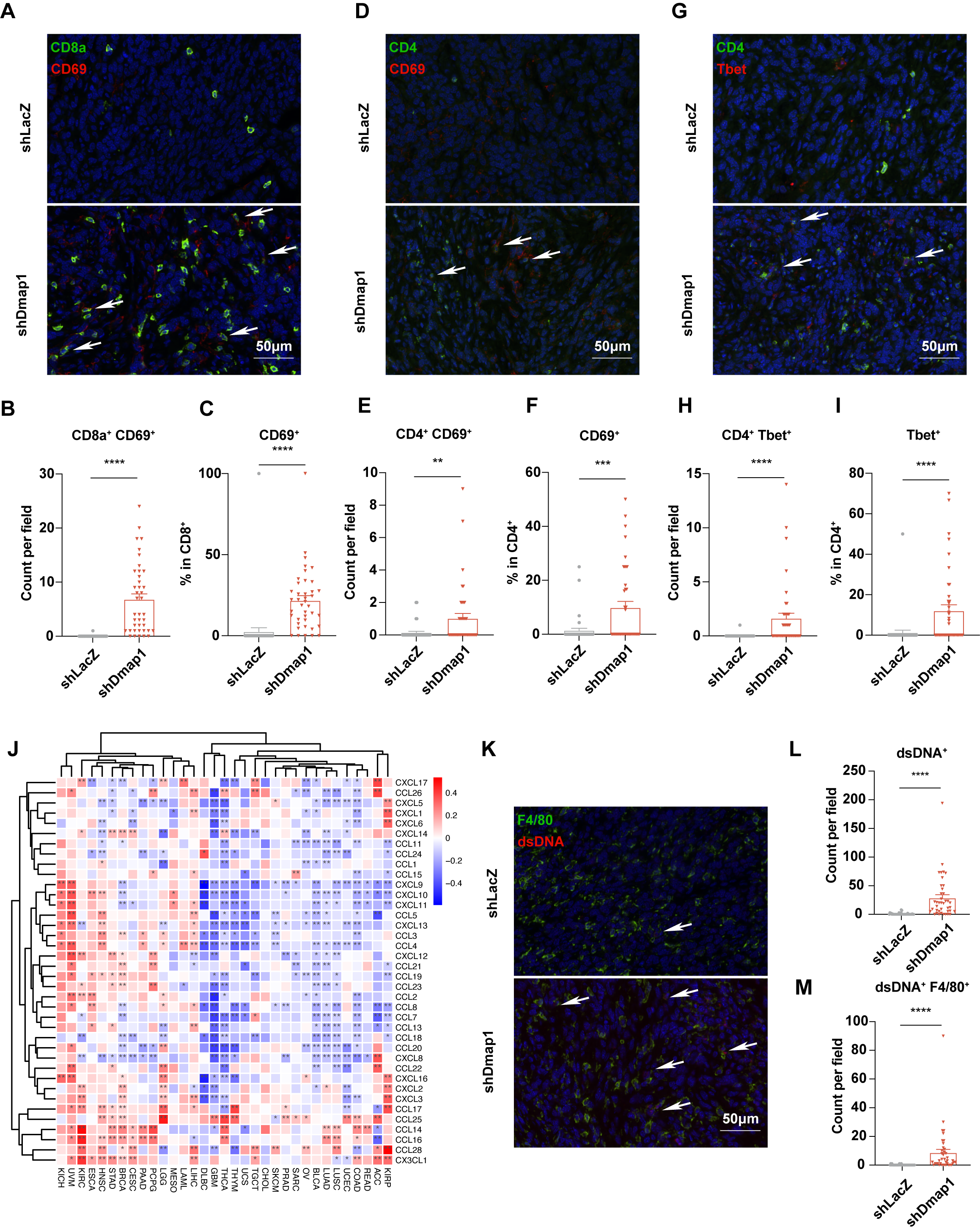


**Figure S7. Dmap1 knockdown stimulates T cell activation and dsDNA generation in the tumor microenvironment. A,** Representative fields of CD8a/CD69 double-positive cells. **B-C**, Statistical analysis for (**A**), Mann-Whitney test, n = 40. **D**, Representative fields of CD4/CD69 double-positive cells. **E-F**, Statistical analysis for (**D**), Mann-Whitney test, n = 40. **G**, Representative fields of CD4/T-bet double-positive cells. **H-I**, Statistical analysis for (**G**), Mann-Whitney test, n = 40. **J,** Heatmap shows the Spearman correlation of DMAP1 expression versus canonical cytokines expression in TCGA cohorts. **K**, Representative fields of F4/80/dsDNA double-positive cells. **L-M**, Statistical analysis for (**K**), Mann-Whitney test, n = 40. *, P < 0.05; **, P < 0.01; ***, P < 0.001; ****, P < 0.0001.


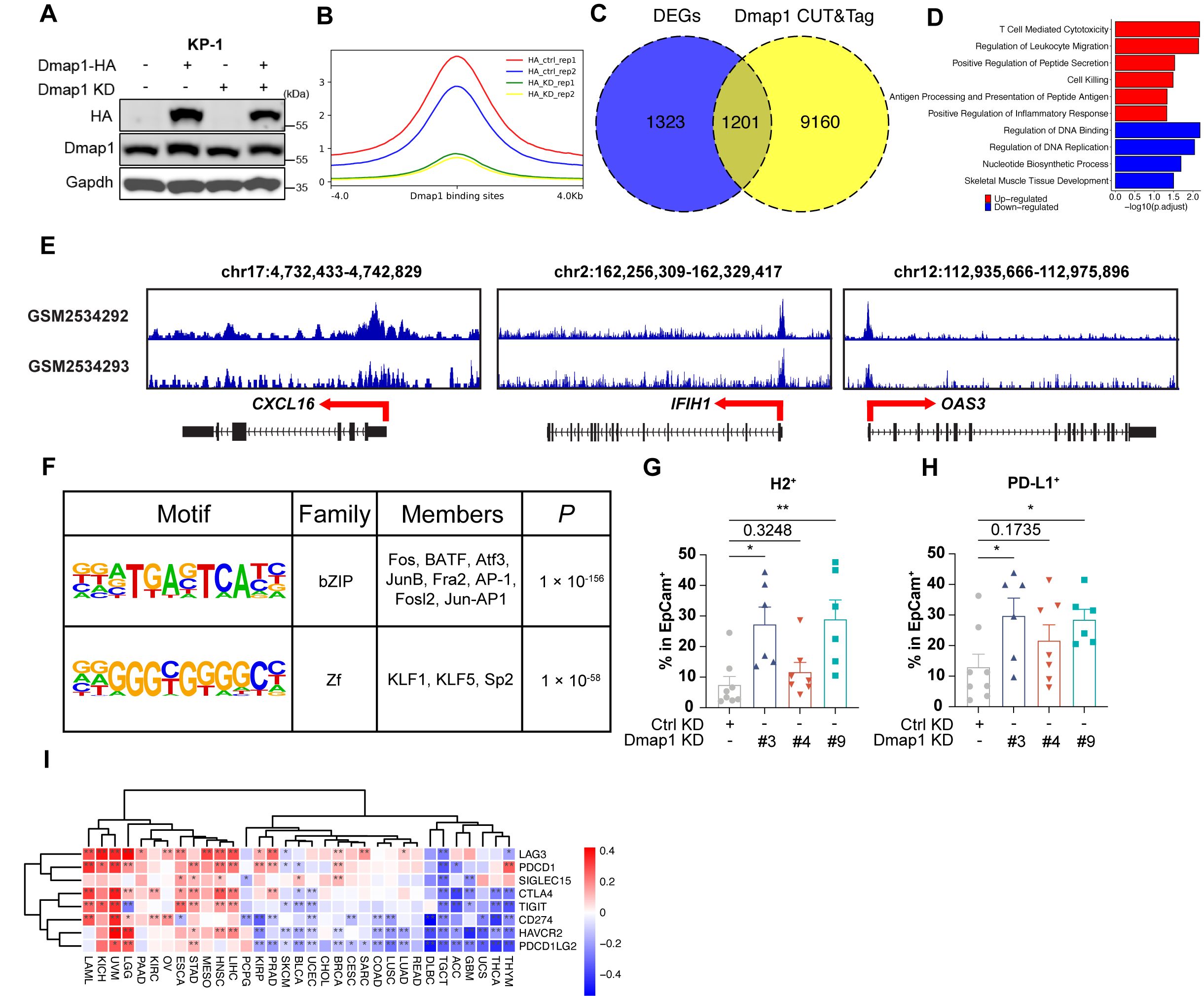


**Figure S8. Dmap1 knockdown upregulates IFN signaling, antigen presentation, and immune checkpoint expression. A**, Western blot for HA-Dmap1 constructs used for CUT&Tag in KP-1 cells. **B**, The binding pattern of Dmap1 in KP-1 Ctrl KD and Dmap1 KD cells. **C**, Venn diagram shows the overlaps of DEGs and Dmap1 direct targets obtained from CUT&Tag results. **D**, The 1,201 DEGs described in (**C**) were functionally enriched in GO gene sets. **E**, Genome browser tracks of HepG2 DMAP1 ChIP-seq data at genomic loci of *CXCL16*, *IFIH1*, and *OAS3.* **F**, HOMER DNA motif analysis in Dmap1 CUT&Tag peaks showing the significant enrichment of Fos and JunB motif, hypergeometric test was used. **G-H**, Flow analysis of H2+ (**G**) and PD-L1+ (**H**) populations in EpCam^+^ tumor cells in the KP-1 C57BL/6 subcutaneous tumors, t-test was used. n = 8 (Ctrl KD), 6 (shDmap1#3), 7 (shDmap1#4), and 6 (shDmap1#9). **I**, Heatmap shows the Spearman correlation of DMAP1 expression versus immune checkpoint molecules in TCGA cohorts. *, P < 0.05; **, P < 0.01.

**
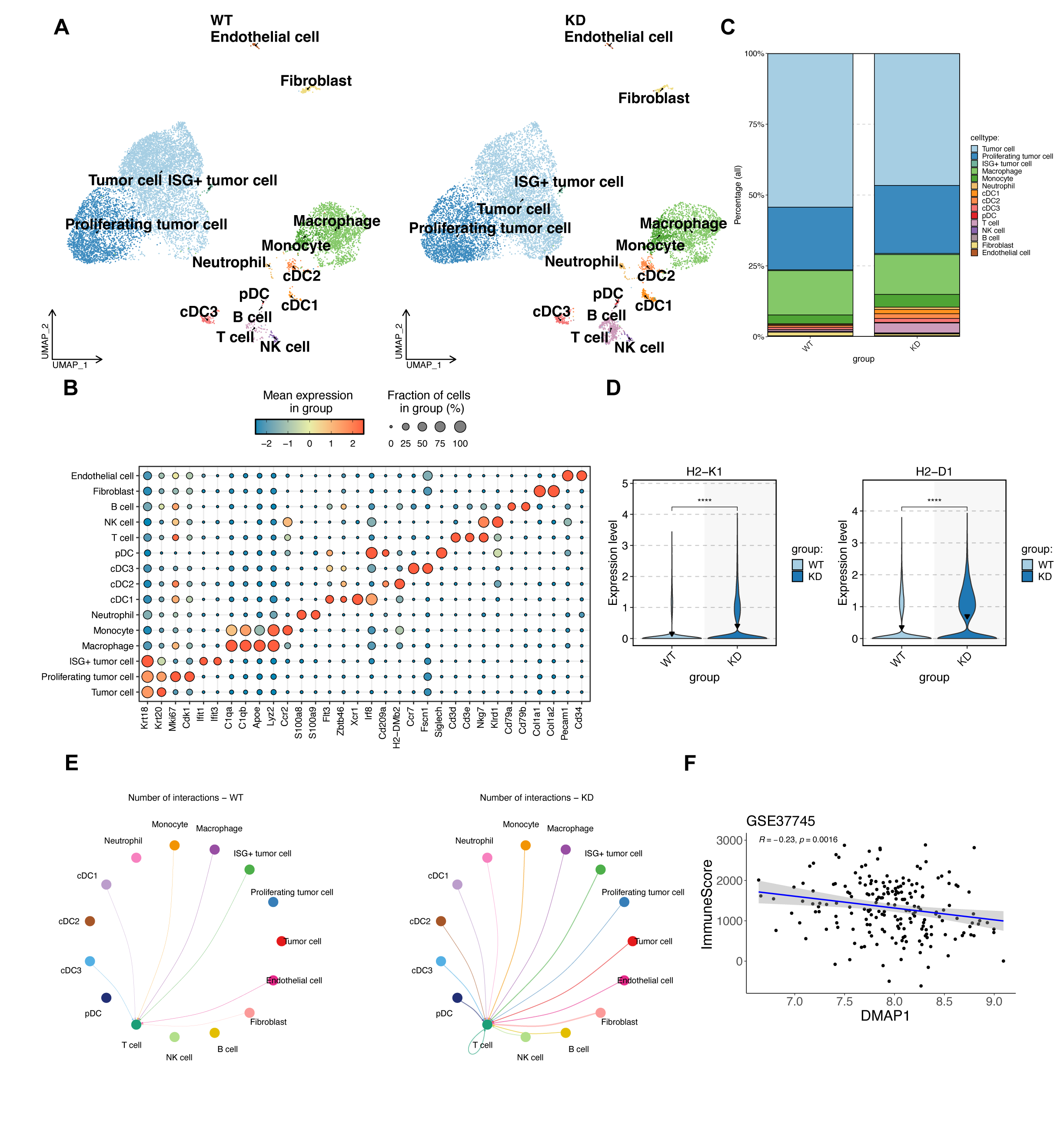
**

**Figure S9. Single-cell analysis of intratumoral immune cell confirms the alteration of T cells. A**, UMAP plot shows clusters of tumor cells and intratumoral immune cells of Ctrl KD and Dmap1 KD tumors. **B**, Heatmap shows the expression of marker genes of different cell clusters. **C**, Changes in different cell compartments in response to Dmap1 knockdown. **D**, Changes in expression levels of MHC I molecules in tumor cells in response to Dmap1 knockdown, the inverted triangle represents the median level. **E**, Circle plot shows cell-cell interactions between T cells and other cell types. **F**, Scatterplot shows the Spearman correlation of DMAP1 expression versus ESTIMATE immune score in the GSE37745 cohort. Data were analyzed using Wilcoxon rank-sum test [(D)]. ****, P < 0.0001.

**
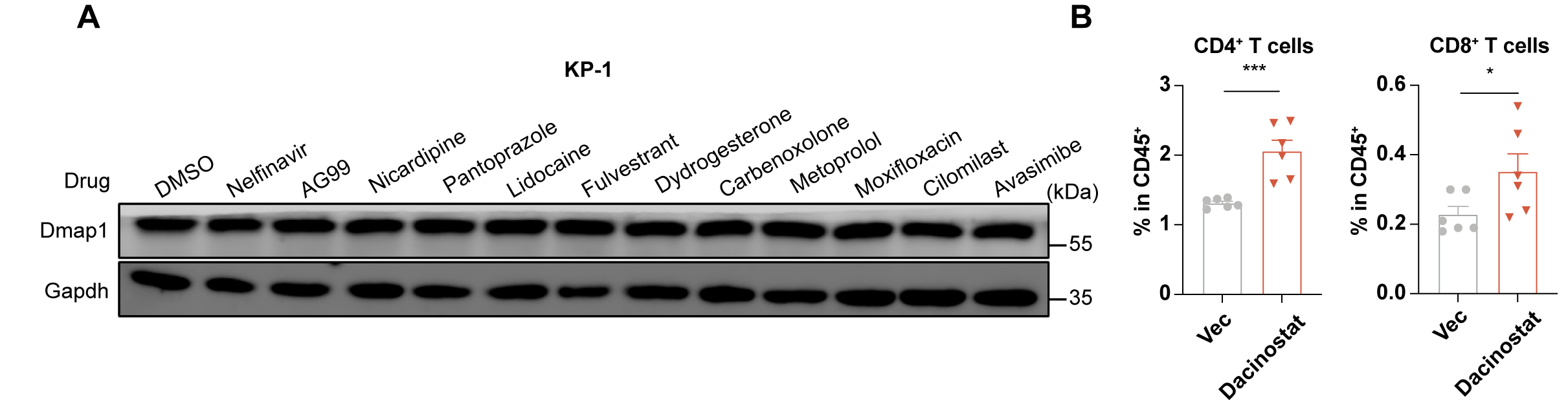
**

**Figure S10. Dmap1 inhibitor exerts immune-mediated antitumor activity *in vivo*.** **A,** Western blot analysis of Dmap1 expression in KP-1 cells treated with 12 candidate Dmap1 inhibitors. cells were exposed to 10μM of each drug for 48h. **B,** Flow cytometry analysis of CD4^+^ (left) and CD8^+^ (right) T cells in CD45^+^ immune cells, n = 6. Data are presented as mean ± SEM. Data were analyzed using two-tailed t-test. *, P < 0.05; ***, P < 0.001.

**Table S1. Target sequences of small hairpin RNA used for shRNA knockdown**

| Name | Species | Target sequence | Type |
| --- | --- | --- | --- |
| shDmap1#3 | Mus musculus | TGCTTTGCTTTACTCTGACAA | small hairpin RNA |
| shDmap1#4 | Mus musculus | GCGCAGATGTACGAGACATTC | small hairpin RNA |
| shDmap1#9 | Mus musculus | GCTGGTGCATATGTTCAATGA | small hairpin RNA |
| shDMAP1#5 | Homo sapiens | GCTGGTGCACATGTTCAATGA | small hairpin RNA |
| shDMAP1#9 | Homo sapiens | GGATGTACGGGACATTCTAGA | small hairpin RNA |
| shLacZ | NT control | CGGATTCTCTGGCCGTCGTAT | small hairpin RNA |
| shGFP | NT control | CAAGCTGACCCTGAAGTTCAT | small hairpin RNA |

**Table S2. Primer sequences used for qRT-PCR**

| Gene name | Species | Direction | Sequence |
| --- | --- | --- | --- |
| Cxcl10 | Mus musculus | Forward | TTCTGAAAGGTGACCAGCCG |
| Cxcl10 | Mus musculus | Reverse | GTCGCACCTCCACATAGCTT |
| Cxcl16 | Mus musculus | Forward | TAGGTGACCTCGTCCCAACA |
| Cxcl16 | Mus musculus | Reverse | CCACACACGCTTTTGGACTG |
| Oas1a | Mus musculus | Forward | ACTTCCAGCAAGCCTGATCC |
| Oas1a | Mus musculus | Reverse | CCCTTGTTCCCAGGCAAAGA |
| Oas3 | Mus musculus | Forward | GGACCATCAACTACAGCGCA |
| Oas3 | Mus musculus | Reverse | TTCACACAGCGGCCTTTACC |
| Oas1c | Mus musculus | Forward | GCCTGTGCAGAGGTCTGATT |
| Oas1c | Mus musculus | Reverse | GGCACCTTGGAAGCATCTCT |
| Ifih1 | Mus musculus | Forward | ATCTGTGGGTGGAAGGCAAT |
| Ifih1 | Mus musculus | Reverse | TACCAAGTAGCCTCCGAGCA |
| Dmap1 | Mus musculus | Forward | AGAGACGCTGACCTTCAAGAGG |
| Dmap1 | Mus musculus | Reverse | TTCGCCTTCACTGTCCGATACC |
| Actb | Mus musculus | Forward | CTGTCCCTGTATGCCTCTG |
| Actb | Mus musculus | Reverse | ATGTCACGCACGATTTCC |

**Table S3. Primer sequences used for CUT&Tag qRT-PCR**

| Target name | Species | Direction | Sequence |
| --- | --- | --- | --- |
| Spike-in | / | Forward | GCCTTCTTCCCATTTCTGATCC |
| Spike-in | / | Reverse | CACGAATCAGCGGTAAAGGT |
| Cxcl10 | Mus musculus | Forward | GACTTCCCTCGGGTTGCG |
| Cxcl10 | Mus musculus | Reverse | GTTGGCTCGGGATGTCTCTC |
| Cxcl16 | Mus musculus | Forward | ACAGGTCTGGTGACTTGTTTG |
| Cxcl16 | Mus musculus | Reverse | TCATCTGTCTGTCTGCTGGTT |
| Oas3 | Mus musculus | Forward | GCACAAGAGCTGGGCAGATA |
| Oas3 | Mus musculus | Reverse | GAAGCTGCTGTGGGTTAGGT |
| Ifih1 | Mus musculus | Forward | TTCTCACGGTGCTCTTGACC |
| Ifih1 | Mus musculus | Reverse | TTCCACCCACAGATCGGTTT |

**References**

[1] F. Li, W.-L. Ng, T. A. Luster, H. Hu, V. O. Sviderskiy, C. M. Dowling, K. E. R. Hollinshead, P. Zouitine, H. Zhang, Q. Huang, M. Ranieri, W. Wang, Z. Fang, T. Chen, J. Deng, K. Zhao, H.-C. So, A. Khodadadi-Jamayran, M. Xu, A. Karatza, V. Pyon, S. Li, Y. Pan, K. Labbe, C. Almonte, J. T. Poirier, G. Miller, R. Possemato, J. Qi, K.-K. Wong, Cancer Res. **2020**, 80, 3556.
